# Multiomics of HER2-low triple-negative breast cancer identifies a receptor tyrosine kinase–relevant subgroup with therapeutic prospects

**DOI:** 10.1172/jci.insight.172366

**Published:** 2023-11-22

**Authors:** Lie Chen, Cui-Cui Liu, Si-Yuan Zhu, Jing-Yu Ge, Yu-Fei Chen, Ding Ma, Zhi-Ming Shao, Ke-Da Yu

**Affiliations:** 1 Department of Breast Surgery, Shanghai Cancer Center and Cancer Institute, Shanghai Medical College, Fudan University, Shanghai, China.; 2 Key Laboratory of Breast Cancer in Shanghai, Shanghai, China.

**Keywords:** Oncology, Breast cancer

## Abstract

To provide complementary information and reveal the molecular characteristics and therapeutic insights of HER2-low breast cancer, we performed this multiomics study of hormone receptor–negative (HR^–^) and HER2-low breast cancer, also known as HER2-low triple-negative breast cancer (TNBC), and identified 3 subgroups: basal-like, receptor tyrosine kinase–relevant (TKR), and mesenchymal stem–like. These 3 subgroups had distinct features and potential therapeutic targets and were validated in external data sets. Interestingly, the TKR subgroup (which exists in both HR^+^ and HR^–^ breast cancer) had activated HER2 and downstream MAPK signaling. In vitro and in vivo patient-derived xenograft experiments revealed that pretreatment of the TKR subgroup with a tyrosine kinase inhibitor (lapatinib or tucatinib) could inhibit HER2 signaling and induce accumulated expression of nonfunctional HER2, resulting in increased sensitivity to the sequential HER2-targeting, Ab–drug conjugate DS-8201. Our findings identify clinically relevant subgroups and provide potential therapeutic strategies for HER2-low TNBC subtypes.

## Graphical abstract

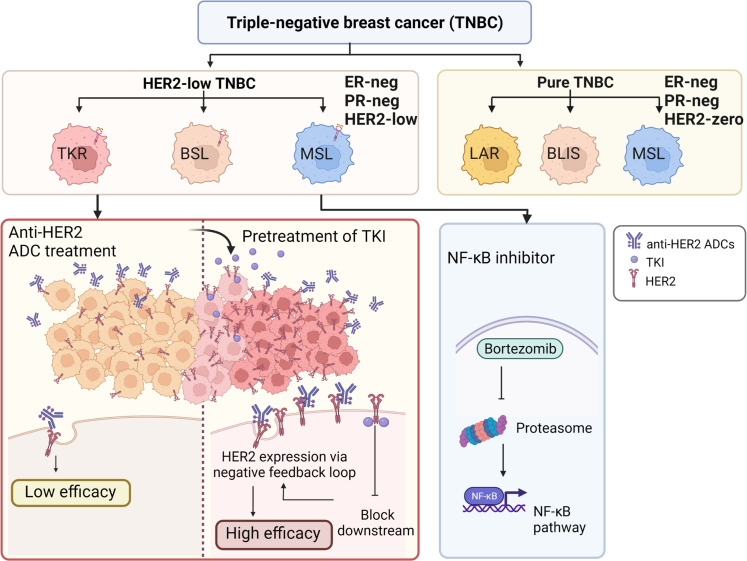

## Introduction

*ERBB2*, a characteristic oncogene (1), is one of the highest-response therapeutic targets in breast cancer. Treatment strategies mainly act on a specific cluster of tumors with *ERBB2* gene amplification and subsequent HER2 protein overexpression (2). This specific cluster is defined as the HER2^+^ subgroup, and the classification criteria are an IHC score of 3+ or of 2+ with ISH positivity (3).

In contrast, breast cancers with low or moderate expression of HER2 without amplification are currently not targetable with conventional anti-HER2 agents. However, an in-depth clinical trial of therapy targeting HER2 has provided some inspiring results showing that trastuzumab deruxtecan, also known as T-DXd and DS-8201 (4), and trastuzumab duocarmazine (SYD985) (5), which are considered Ab–drug conjugates (ADCs), could have potential clinical value in tumors with low and moderate expression of HER2 (6, 7). Recently, results of DESTINY-Breast04 clinical trials (ClinicalTrials.gov identifier NCT03734029) demonstrated that longer progression-free and overall survival in patients with HER2-low metastatic breast cancer stemmed from trastuzumab deruxtecan (hereafter, DS-8201) (8). These results are challenging for clinical pathology, which focuses mainly on detecting HER2^+^ carcinomas.

The current HER2 testing algorithm can distinguish tumors that are completely negative for HER2 (IHC score 0; HER2-0) from HER2-low tumors (low [IHC 1+] or moderate expression [IHC score 2+; ISH negative]) (9). Because HER2-0 cases have often been combined with HER2-low cases, the clinical and biological understanding of HER2-low tumors is limited. Nevertheless, based on the results of 4 prospective neoadjuvant clinical trials, compared with HER2-0 tumors, HER2-low tumors have differences in biological characteristics, response to therapy, and prognosis (10). Thus, HER2-low tumors represent a subgroup with potential therapeutic value, and precision medicine for this subgroup of tumors might benefit patients with breast cancer, especially those with triple-negative breast cancer (TNBC) for whom therapy strategies are scarce (11, 12).

Our previous TNBC research has focused on molecular subgroups, and an effective method for uncovering possible therapeutic targets has been reported (13). The present study involved a multiomics analysis of patients with HR^–^ and HER2-low breast cancer (hereafter, HER2-low TNBC) from Fudan University Shanghai Cancer Center (FUSCC) and data from The Cancer Genome Atlas (TCGA) to exhibit a broad molecular spectrum of HER2-low TNBC, reveal molecular subgroups, and provide potential clinical relevance. We also conducted molecular and biological experiments to verify the reliability of the molecular subgroups and potential therapeutic targets.

## Results

### Transcriptomic profiling reveals HER2-low subgroups.

**Figure 1:**
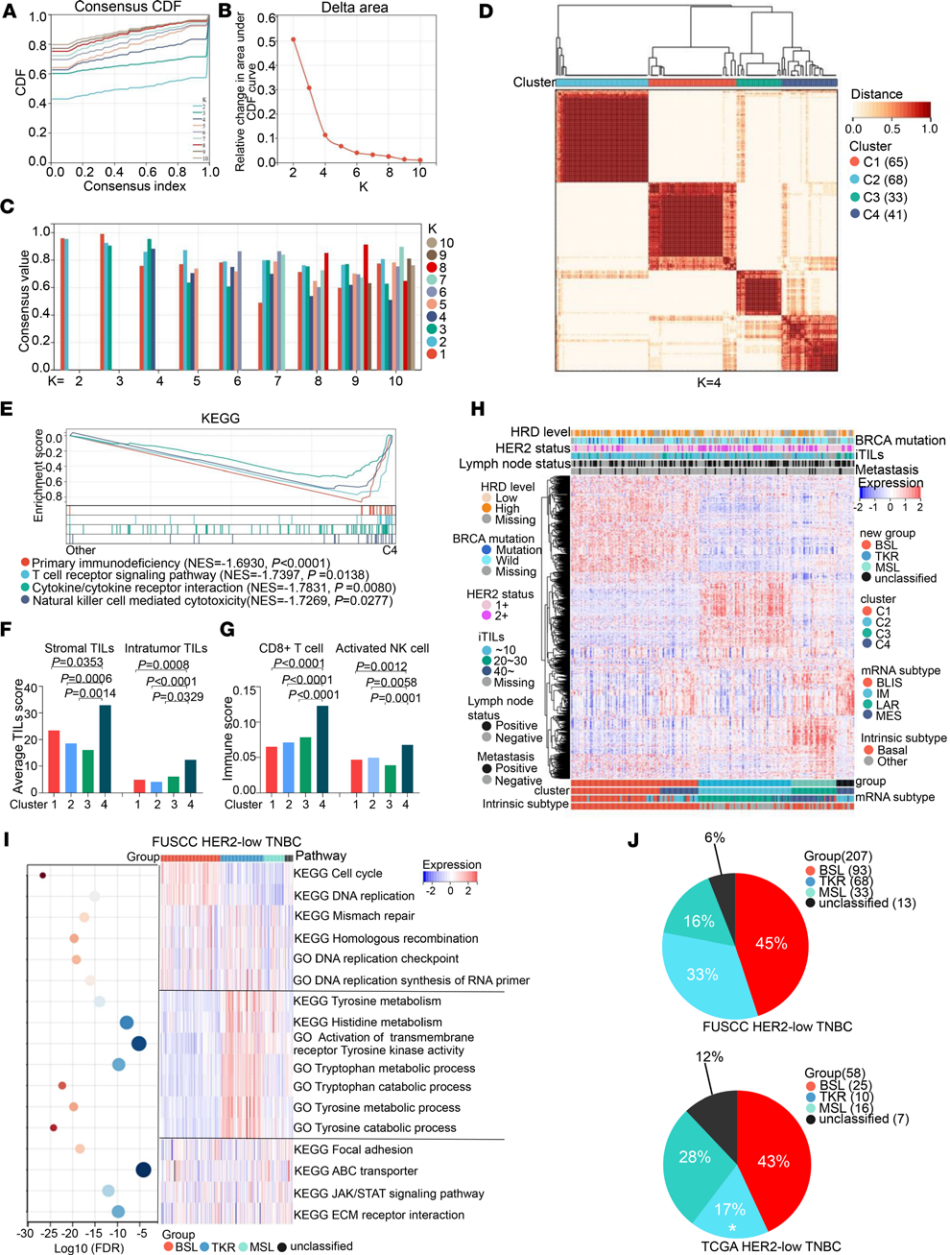
Transcriptomic profiling reveals HER2-low TNBC subgroups. (**A**) Consensus empirical cumulative distribution function (CDF) curves of K = 2–10. (**B**) Delta area changes with K = 2–10. (**C**) Consensus values of different K values. (**D**) Consensus clustering matrices of the 207 HER2-low TNBC samples with mRNA expression at K = 4. (**E**) Gene set enrichment analysis (GSEA) of primary immunodeficiency, T cell receptor signaling pathway, cytokine/cytokine receptor interaction, and NK cell–mediated cytotoxicity between C4 and other clusters based on the KEGG data set (permutation test). (**F**) Average stromal and intratumoral TIL scores in the 4 subgroups (Kruskal-Wallis test followed by Dunn’s multiple comparisons test). (**G**) Relative abundance of 2 immune-activated cells in 4 subgroups based on CIBERSORT (1-way ANOVA followed by Dunnett’s *t* test). (**H**) Heatmap showing the top 2,000 variable mRNAs of 207 HER2-low TNBC samples; clinical and molecular features are annotated. (**I**) ssGSEA of 207 HER2-low TNBC samples based on KEGG and GO data sets are shown in the heatmap, and the FDRs are shown in the bubble plot. (**J**) Distribution of HER2-low TNBC mRNA subgroups in the FUSCC (top) and TCGA (bottom) data sets (χ^2^ test). The samples of **A**–**I** were from HER2-low TNBC based on the FUSCC data set. Statistical significance was set at *P* < 0.05.

We analyzed HER2-low tumors from TNBC samples because there might be heterogeneity between HR^–^ and HR^+^ cases. Identifying subgroups of HER2-low tumors among TNBC cases is meaningful and clinically relevant given the limited strategies for treating TNBC. We chose 207 HER2-low TNBC (IHC score 1+, *n* = 140; IHC score 2+ and ISH negative, *n* = 67) samples from our FUSCC TNBC data set. On the basis of the top 2,000 most variable mRNAs, we performed unsupervised partitioning around medoid clustering to preliminarily classify the samples. According to related consensus empirical cumulative distribution function curves (Figure 1, A and B) and consensus values (Figure 1C) of different K values (from 2 to 10), 4 clusters of HER2-low TNBC samples were identified (Figure 1D and Supplemental Figure 1A; supplemental material available online with this article; https://doi.org/10.1172/jci.insight.172366DS1; clusters 1–4 are denoted C1, C2, C3, and C4, respectively). GSEA showed that primary immunodeficiency (normalized enrichment score [NES] = –1.6930; *P* < 0.0001), T cell receptor signaling pathway (NES = –1.7397; *P* = 0.0138), cytokine and cytokine receptor interactions (NES = –1.7831; *P* = 0.0080), and NK cell–mediated cytotoxicity (NES = –1.7269; *P* = 0.0277) were significantly different between C4 and the other clusters (Figure 1E). Furthermore, H&E staining demonstrated a high level of both stromal tumor-infiltrating lymphocytes (TILs; C4 vs. C1, *P* = 0.0353; vs. C2, *P* = 0.0006; vs. C3, *P* = 0.0014) and intratumor TILs (C4 vs. C1, *P* = 0.0008; vs. C2, *P* < 0.0001; vs. C3, *P* = 0.0329) in C4 (Figure 1F), and a CIBERSORT analysis (14) illustrated that several immune-activated cells (15), such as CD8^+^ T cells (C4 vs. C1, *P* < 0.0001; vs. C2, *P* < 0.0001; vs. C3, *P* < 0.0001) and activated NK cells (C4 vs. C1, *P* = 0.0012; vs. C2, *P* = 0.0058; vs. C3, *P* = 0.0001), were relatively enriched in C4 (Figure 1G).

These results suggested that C4 was an immune-related cluster. The immune-related features were contributed by immune cells rather than tumor cells (16), and immune cells consisted of various tumor subgroups, so these features could be used to describe tumor immunity conditions, not an independent cluster (17). Therefore, we regrouped 207 samples (Figure 1H) and performed single-sample GSEA (ssGSEA) to identify enriched scores based on gene sets from different signaling pathways and then distinguish the characteristic of each subgroup (Figure 1I and Supplemental Figure 1B).

The results showed that the cell cycle and DNA replication scores were higher in 1 subgroup, which we identified as a basal-like (BSL) subgroup; several aa metabolism (tyrosine, histidine, and tryptophan) and receptor tyrosine kinase activity scores, but not androgen receptor (AR) signaling pathway scores, were higher in another subgroup, which we identified as a receptor tyrosine kinase–relevant (TKR) subgroup; whereas stem cell–related pathways, including extracellular matrix–receptor interactions, focal adhesion, and ATP-binding cassette transporters, were higher in the next subgroup, so we regarded it as a mesenchymal stem–like (MSL) subgroup. Similar results were observed in TCGA data set (Supplemental Figure 1, B and C).

As mentioned, we divided the HER2-low TNBC samples from FUSCC into 3 main subgroups, namely, BSL (*n* = 93; 45%), TKR (*n* = 68; 33%), and MSL (*n* = 33; 16%), and an unclassified subgroup (*n* = 13; 6%) (Figure 1J, top). TCGA data set was used to verify these groupings, and the results illustrated HER2-low TNBC samples in TCGA could be further divided into BSL (*n* = 25; 43%), TKR (*n* = 10; 17%), MSL (*n* = 16; 28%), and unclassified (*n* = 7; 12%) subgroups (Figure 1J, bottom). Among TCGA HER2-low TNBC samples, the TKR subgroup was more prevalent among the FUSCC samples (33% FUSCC vs. 17% TCGA; *P* = 0.0212).

To verify whether HER2 status exerted a definite influence on our subgroups, we compared the proportions of the 4 subgroups in both HER2 IHC score 1+ and HER2 IHC score 2+ samples (Supplemental Figure 1D). Compared with those in the HER2 1+ samples, the proportions of the TKR and BSL subgroups were higher and lower, respectively, in the HER2 2+ samples (51% vs. 24%, *P* < 0.05; 25% vs. 54%, *P* < 0.05). These results indicated that HER2 status plays a crucial role in the subgrouping of HER2-low TNBC.

We then conducted immune microenvironment analyses to explore the immune features among the 3 major subgroups. H&E staining demonstrated that although there was a distinction in stromal TILs among the BSL, TKR, and MSL subgroups (*P* = 0.0061), there was no significant difference in intratumor TILs among the subgroups (Supplemental Figure 2A).

We performed a CIBERSORT analysis (14) of the relative abundance of 22 kinds of immune cells in HER2-low TNBC samples from FUSCC (Supplemental Figure 2B) and found that neither the relative abundance of immune-activated cells (15), such as CD8^+^ T cells, activated NK cells, or M1 macrophages, nor immune-inhibited cells (15), such as Tregs, were significantly different among the BSL, TKR, and MSL subgroups (Supplemental Figure 2, C and D). Interestingly, as another type of immune-inhibited cell, the relative abundance of M2 macrophages was diverse (*P* < 0.0001; Supplemental Figure 2D). For TCGA data set (Supplemental Figure 2E), the results showed that there was no significant difference in the relative abundance of the abovementioned immune-activated cells or immune-inhibited cells among the 3 subgroups (Supplemental Figure 2, F and G). These results indicated that the impact of the main immune cells was evenly distributed in the 3 subgroups.

We further examined the HER2-low subgroups in HR^+^ samples and found TKR, BSL, and luminal (LUM) subgroups. The major subgroup was the LUM subgroup (*n* = 164 [65%]; Supplemental Figure 3, A and B), which had enriched gene sets of relative estrogen receptor signaling pathways (Supplemental Figure 3C). The TKR subgroup was also present among the HR^+^ and HER2-low TNBC samples based on TCGA data set (*n* = 51; 20%) (Supplemental Figure 3, A and B) and exhibited enrichment of gene sets from receptor tyrosine kinase activity, including ERBB and ERBB2 signaling pathways (Supplemental Figure 3C). The proportions of the BSL (*n* = 21) and unclassified (*n* = 15) subgroups were 8% and 6%, respectively (Supplemental Figure 3, A and B). These results revealed that the TKR subgroup was present not only in HR^–^ breast cancer but also in HR^+^ HER2-low TNBC.

### HER2-low subgroups based on multiomics data have distinct features.

**Figure 2:**
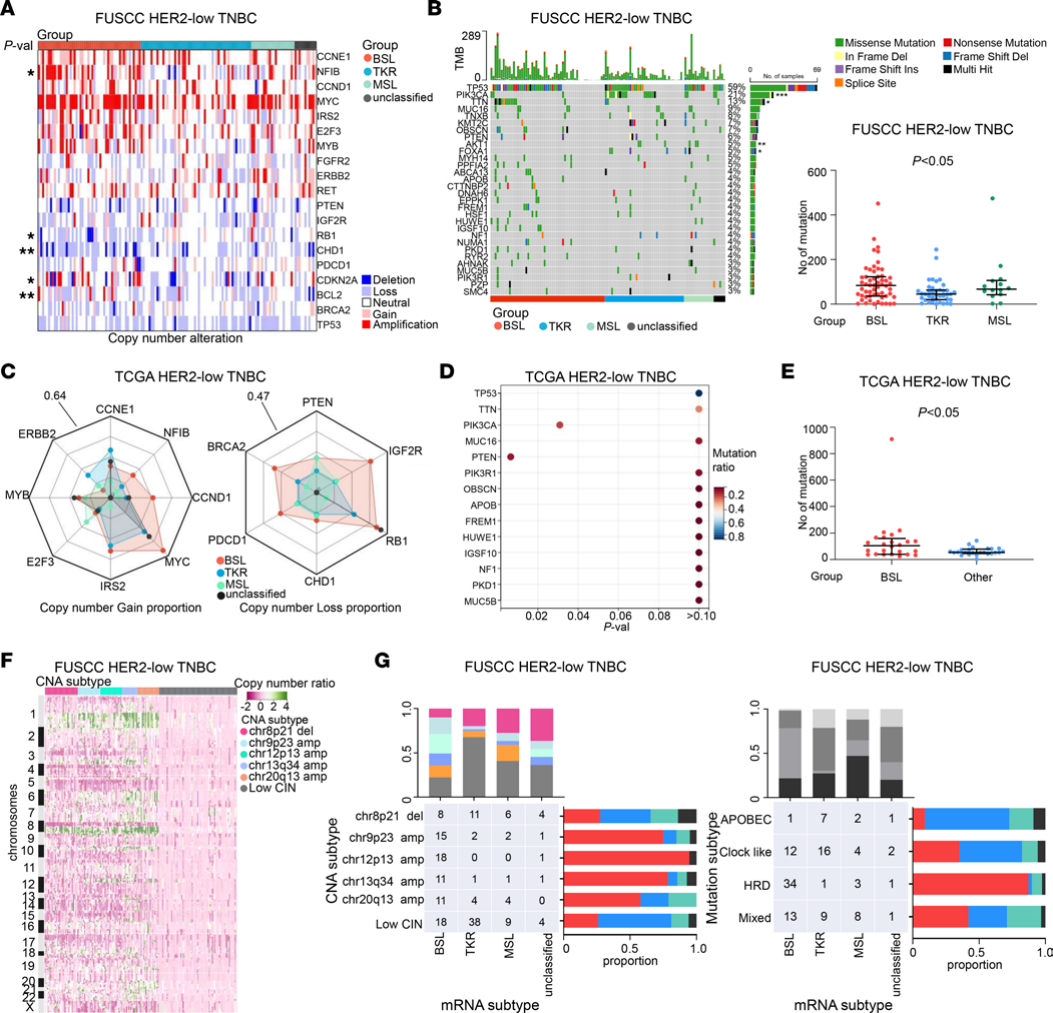
HER2-low TNBC subgroups based on multiomics data have distinct features. (**A**) Specific oncogenes and tumor suppressor genes (TSGs) with top-rank GISTIC peaks. (**B**) The top 30 genes with the most frequent mutations (at least 3% of the cases; left) and total mutation numbers among the BSL, TKR, and MSL subgroups (right; Kruskal-Wallis test). (**C**) Radar chart illustrating the proportion of copy number gain (left) and loss (right) of specific oncogenes and TSGs. (**D**) Genes with the most frequent mutations (at least 3% of the cases). (**E**) Total mutation numbers between BSL and other groups (Mann-Whitney test). (**F**) Copy number–based clustering on the basis of GISTIC peaks is shown in the heatmap. (**G**) Relationships between mRNA subgroups and CNA subtypes (left) and mRNA subgroups and mutation subtypes (right). The samples for the data reported in **A**, **B**, **F**, and **G** were from FUSCC data set; those for **C**–**E** were from TCGA data set. Statistical significance was set at *P* < 0.05. *P < 0.05, **P < 0.01, ***P < 0.001. CIN, low chromosomal instability; Del, deletion; Ins, insertion; Multi, multiple; Val, value.

Subsequently, we examined specific oncogenes and tumor suppressor genes with top-rank Genomic Identification of Significant Targets in Cancer (GISTIC) peaks to estimate somatic copy number alterations (CNAs) in HER2-low TNBC samples from FUSCC. *CCNE1*, *NFIB*, *CCND1*, *MYC*, *IRS2*, *E2F3*, *MYB*, *FGFR2*, *ERBB2*, and *RET* exhibited frequent gains, whereas *TP53*, *BRCA2*, *BCL2*, *CDKN2A*, *CHD1*, *RB1*, *IGF2R*, and *PTEN* exhibited frequent losses (Figure 2A). Interestingly, *PDCD1* (encoding programmed cell death protein 1), which is considered an immune-inhibitory receptor (18) and provides a new and reliable direction for cancer immunotherapy (19), was also frequently affected by CNAs in HER2-low TNBC samples (Figure 2A). Similar results were found in TCGA data set, as illustrated in the radar charts in Figure 2C.

We also assessed somatic mutations in the HER2-low TNBC samples from the FUSCC data set. The top 5 most frequent variations were *TP53* (59%), *PIK3CA* (21%), *TTN* (13%), *MUC16* (9%), and *TNXB* (8%) (Figure 2B, left). The mutation rates of *PIK3CA*, *TTN*, *AKT1*, and *FOXA1* were distinct among our subgroups of HER2-low TNBC (Figure 2B, left). The *PIK3CA* and *PTEN* mutation rates were statistically significantly different among the subgroups from TCGA data set (Figure 2D). We further calculated the total mutation numbers of the HER2-low TNBC samples. As shown in the graph in Figure 2B, there was a difference in total mutation numbers among the BSL, TKR, and MSL subgroups based on the FUSCC data set, and TCGA data indicate BSL had more total mutation numbers than the other subgroups did (Figure 2E).

Six CNA-related subgroups were identified in our previous FUSCC research (13): chr8p21 del (*n* = 29; 17%), chr9p23 amp (*n* = 20; 12%), chr12p13 amp (*n* = 19; 11%), chr13q34 (*n* = 14; 8%), chr20q13 (*n* = 19; 11%), and low chromosomal instability (*n* = 69; 41%) (Figure 2F and Supplemental Figure 4A). Four mutation-relevant clusters were also identified by using the following mutational signatures from the Catalog of Somatic Mutations in Cancer (https://cancer.sanger.ac.uk/cosmic): APOBEC (*n* = 11; 10%), homologous recombination deficiency (HRD; *n* = 34; 29%), clock-like (*n* = 39; 34%), and mixed (*n* = 31; 27%) (Supplemental Figure 4B). Then, we analyzed the potential relationships among expression, CNA, and mutation-relevant subgroups (Figure 2G and Supplemental Figure 4C). For example, the BSL subgroup was the majority in the chr12p13 amp (95%) and HRD (87%) subgroups; the TKR subgroup was the majority in the low chromosomal instability subgroup (55%) and the minority in the HRD subgroup (3%).

Several characteristics of the HER2-low TNBC subgroups, including clinical information, mutation status, and potential signaling pathway conditions, are listed in Supplemental Table 1, and indicate the application of potential diagnostic methods and precision therapeutic targets for these subgroups.

### Activated ERBB2-mediated receptor tyrosine kinase in the TKR subgroup.

**Figure 3:**
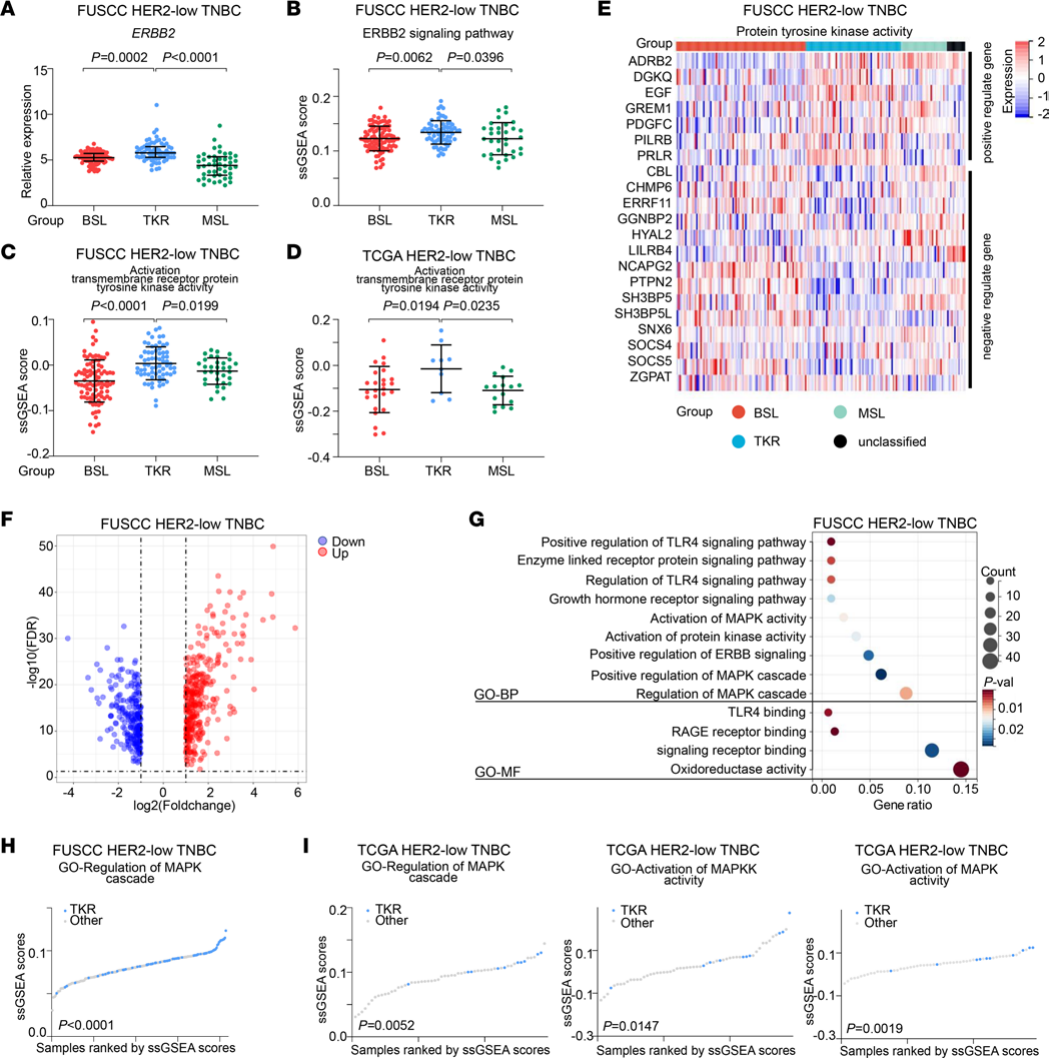
Activated *ERBB2*-mediated receptor tyrosine kinase in the TKR subgroup. (**A**) Relative expression of *ERBB2* among the BSL, TKR, and MSL subgroups (Kruskal-Wallis test followed by Dunn’s multiple comparisons test). (**B**) GO-ERBB2 signaling pathway ssGSEA scores among the BSL, TKR, and MSL subgroups. (**C** and **D**) GO activation transmembrane receptor protein tyrosine kinase activity ssGSEA scores among the BSL, TKR, and MSL subgroups from the FUSCC (**C**) and TCGA (**D**) data sets. (**B**–**D**) One-way ANOVA followed by Dunnett’s *t* test. (**E**) Expression levels of protein tyrosine kinase activity–related genes across the mRNA subgroups (upper, positively regulated genes; lower, negatively regulated genes). (**F**) Volcano plot illustrating DEGs between the TKR subgroup and the other subgroups. (**G**) GO Biological Process (GO-BP) and GO Molecular Function (GO-MF) pathways were enriched on the basis of upregulated DEGs in the TKR subgroup (hypergeometric test). (**H**) GO regulation of MAPK cascade ssGSEA scores between TKR and other subgroups in HER2-low TNBC from the FUSCC data set. (**I**) GO regulation of MAPK cascade (left), GO activated MAPKK activity ssGSEA scores (middle), and GO activated MAPK activity ssGSEA scores (right) between TKR and other subgroups in HER2-low TNBC from the TCGA data set. (**H** and **I**) Student’s *t* test. Statistical significance was set at *P* < 0.05.

We then uncovered the molecular characteristics and therapeutic targets of the TKR subgroup. The *ERBB2* expression of the TKR subgroup was higher than those of the BSL (*P* = 0.0002) and MSL (*P* < 0.0001) subgroups in samples from the FUSCC data set (Figure 3A). Furthermore, gene ontology–ERBB2 (GO-ERBB2) signaling pathway ssGSEA scores (20) in the TKR subgroup were higher than those in the BSL (*P* = 0.0062) and MSL (*P* = 0.0396)subgroups (Figure 3B). Both in the FUSCC (Figure 3C) and TCGA (Figure 3D) data sets, the GO activation of transmembrane receptor tyrosine kinase activity ssGSEA scores for the TKR subgroup were higher than those for the other 2 subgroups. There was a strong positive correlation between *ERBB2* expression and ERBB2 signaling pathway ssGSEA scores (Supplemental Figure 5A, left). ERBB2 signaling pathway–related gene expression in the TKR and other subgroups is shown in Supplemental Figure 5B. *PTK6*, *PTPRR*, and *STUB1* expression levels were higher in the TKR subgroup than in the other subgroups (Supplemental Figure 5B).

*ERBB2* encodes HER2, which is considered a receptor tyrosine kinase (21, 22). We further analyzed the correlation between the ERBB2 signaling pathway and activation of transmembrane receptor tyrosine kinase activity ssGSEA scores (Supplemental Figure 5A, right), which suggested that the activation of transmembrane receptor tyrosine kinase activity was positively correlated with the activation of *ERBB2*. Regulation of protein tyrosine kinase activity was also distinct between the TKR subgroup and the other subgroups. On the basis of GO-negative and GO-positive regulation of protein tyrosine kinase activity, we calculated the corresponding ssGSEA scores. These scores revealed that the TKR subgroup samples had enriched gene sets of relative protein tyrosine kinase activity, which might suggest activation of protein tyrosine kinase activity (Supplemental Figure 5C). The expression levels of the main genes that participate in the regulation of protein tyrosine kinase activity are shown in Figure 3E. Epidermal growth factor (*EGF*) expression was upregulated in the TKR subgroup, which could stem from less loss and deletion of *EGF* copy numbers (Supplemental Figure 5D).

### Activation of the HER2-MAPK pathway in the TKR subgroup and treatment relevance.

To further explore the key transcriptomic signatures in the TKR subgroup, we compared the differences in the whole transcriptome between the TKR subgroup and other subgroups, based on the FUSCC data set. A total of 629 differentially expressed genes (DEGs) (up: *n* = 374; down: *n* = 255) are shown in Figure 3F. For the HER2-low TNBC samples, the GO cluster plots illustrated that the upregulated DEGs in the TKR subgroup were enriched in MAPK-related pathways, such as activation of MAPK activity, regulation of the MAPK cascade, and positive regulation of the MAPK cascade (Figure 3G). Regulation of the MAPK cascade ssGSEA scores were higher (*P* < 0.0001) in the TKR subgroup than in other subgroups from the FUSCC data set (Figure 3H). As external validation data, regulation of the MAPK cascade, activation of MAPK kinase (MAPKK) activity, and MAPK activity in TCGA data set are shown in Figure 3I. The activation of MAPKK activity (*P* = 0.0147) and activation of MAPK activity (*P* = 0.0019) ssGSEA scores were higher in the TKR subgroup than in the other subgroups.

HR^+^ and HER2-low TNBC samples based on TCGA data set were another set of external validation data. The results revealed that phospho-BRAF (phospho-BRAF, MAPKKK, *P* = 0.0441) (23), phospho-MEK1 (phospho-MEK1, MAPKK, *P* = 0.0002), and p-MAPK (*P* = 0.0002) were present at high levels in the TKR subgroup (Supplemental Figure 5E). The MAPK signaling pathway gene set was enriched in the TKR subgroup (*P* = 0.0322), based on the Kyoto Encyclopedia of Genes and Genomes (KEGG) data set (Supplemental Figure 5F). These results showed that the MAPK signaling pathway might be activated in the TKR subgroup of HER2-low TNBC samples, regardless of estrogen receptor status.

To verify whether the HER2-MAPK pathway is a potential target for the TKR subgroup, we then constructed TKR scores on the basis of expression of *ERBB2*, *GRB7*, *ERBB3*, *EGF*, *PDGFB*, *PTPRR*, and *MAP3K1* from gene sets of the ERBB2 signaling pathway (20), ERBB signaling pathway (20), positive regulation of protein tyrosine kinase activity, and MAPK cascade (24). TKR scores were higher in TKR subgroup (*P* < 0.0001; Supplemental Figure 5G, left) and the standard receiver operating characteristic (ROC) AUC was 0.79 (Supplemental Figure 5G, right) on the basis of the FUSCC data set. As external validation data, the results of HER2-low TNBC samples were similar (Supplemental Figure 5H) and the TKR subgroup constituted more of the high-TKR-scores group than did the low-scores group (45 of 154 vs. 16 of 155, respectively; *P* < 0.0001) from total HER2-low TNBC samples regardless of HR status (Supplemental Figure 5I), based on TCGA data set.

We further selected 27 HER2-low TNBC cells on the basis of *ERBB2* expression from the Genomics of Drug Sensitivity in Cancer database (between quartiles was considered HER2-low) (Supplemental Figure 6A). MFM-223 (negative for HR expression) and ZR-75-1 (low and limited HR expression) cells were identified as having TKR features, based on the TKR scores (Supplemental Figure 6B). MAPK phosphorylation levels of MFM-223 and ZR-75-1 cells were above the median level of HER2-low TNBC cells, based on the MD Anderson Cell Lines Project data set (Supplemental Figure 6C). MFM-223 and ZR-75-1 cells were used for additional experiments, representing HER2-low TKR subgroup cell lines.

Lapatinib, pyrotinib, neratinib, and tucatinib are small-molecule tyrosine kinase inhibitors (TKIs) used to treat HER2^+^ breast cancer (25–28). We treated MFM-223 and ZR-75-1 cells with 4 TKIs; SK-BR-3 cells were used as a positive control, and MDA-MB-231 cells were used as a negative control. As expected, moderate inhibitory activity on cell growth was observed in the MFM-223 cells, with IC_50_ values of 39.8 μM for lapatinib, 3.6 μM for pyrotinib, 3.0 μM for neratinib, and 17.8 μM for tucatinib (Supplemental Figure 6D). In contrast, no such inhibition was observed in the MDA-MB-231 cells, with IC_50_ values greater than 100,000 μM for the 4 TKIs (Supplemental Figure 6D). Similarly, ZR-75-1 cells were moderately susceptible to the 4 TKIs (Supplemental Figure 6D). These results indicated that the TKR subgroup of HER2-low TNBC cells may benefit from TKIs.

**Figure 4:**
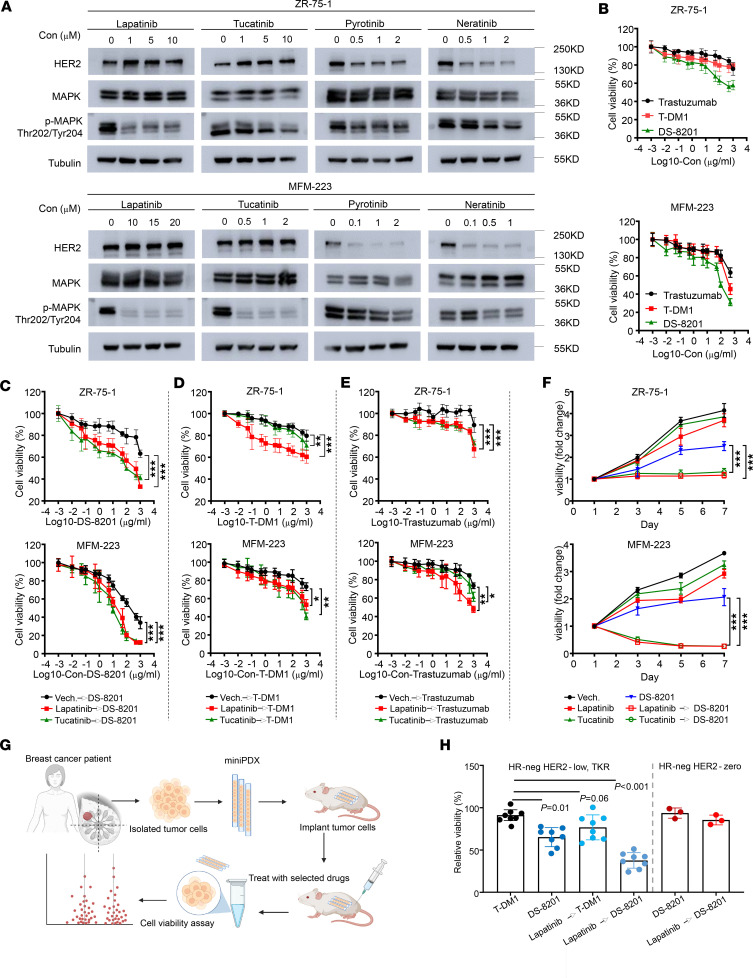
HER2/MAPK pathway activation in the TKR subgroup and treatment relevance. (**A**) Western blot showing HER2, p-MAPK (Thr202/Tyr204), total MAPK, and tubulin (loading control) expression in total lysates of both ZR-75-1 (top) and MFM-223 (bottom) cells treated for 48 hours with lapatinib, tucatinib, pyrotinib, and neratinib at different concentrations. Untreated cells served as controls. (**B**) Cell Counting Kit-8 (CCK-8) assay was used to measure the cell viability of ZR-75-1 (*n* = 7) and MFM-223 (*n* = 6) cells in vitro. The cells were treated with serial concentrations of trastuzumab, T-DM1, and DS-8201 for 48 hours. (**C**) The CCK-8 assay was used to measure cell viability of ZR-75-1 cells (*n* = 7) against lapatinib (1 μM), tucatinib (1 μM), or Vech. for 48 hours and sequential treatment of DS-8201 for 48 hours (top) and MFM-223 cells (*n* = 6) against lapatinib (10 μM), tucatinib (1 μM) or Vech. for 48 hours and sequentially treated DS-8201 for 48 hours. Each point represents the mean and SD. (**D**) Sequential treatment of T-DM1. (**E**) Sequential treatment of trastuzumab. (**F**) CCK-8 assay of ZR-75-1 (top) and MFM-223 (bottom) cells against lapatinib, tucatinib, or Vech. and sequential treatment of DS-8201. Each point represents the mean and SD (*n* = 6). (**G**) Scheme of the generation of the miniPDX models for the in vivo pharmacological tests. (**H**) Relative viability of miniPDX models with different treatment strategies, as normalized to saline treatment. HER2-low TKR group, *n* = 8; pure TNBC (with HER2-0) group, *n* = 3 (1-way ANOVA followed by Dunnett’s *t* test). Data are presented as mean and SD. Statistical significance was set at *P* < 0.05. *P < 0.05, **P < 0.001, ***P < 0.001. Con, concentration; Neg, negative.

Lapatinib-induced accumulation of inactive HER2 leads to increased Ab-dependent cell-mediated cytotoxicity in HER2^+^ breast cancer cells in vitro and in vivo (29). To validate this effect in the TKR subgroup of HER2-low TNBC cells, we treated MFM-223 and ZR-75-1 cells with lower concentrations than the IC_50_ values of the TKIs. Western blotting results showed that all 4 TKIs, at low doses, inhibited MAPK phosphorylation (Thr202/Tyr204), an indicator of inhibition of HER2 signaling when compared with untreated cells (Figure 4A). Interestingly, lapatinib and tucatinib induced the accumulation of total HER2 at low concentrations (Figure 4A). Therefore, we designed a combined sequential therapy for the TKR subgroup with a TKI (lapatinib or tucatinib) and ADCs. The ADCs T-DM1 and DS-8201, comprising an anti-HER2 mAb, a linker, and a cytotoxic agent payload, are used as a targeted therapy for HER2^+^ malignancies (30, 31). Moreover, DS-8201 has been viewed as a promising treatment for patients with breast or gastric cancer expressing low HER2 levels (32). Recently, the DESTINY-Break04 clinical trial illustrated that DS-8201 could significantly prolong the progression-free survival and overall survival in patients with HER2-low metastatic breast cancer (8).

To avoid massive cell death, we first treated MFM-223 and ZR-75-1 cells with low concentrations of lapatinib and tucatinib for approximately 48 hours and then sequentially treated them with serial concentrations of DS-8201 (Figure 4C), T-DM1 (Figure 4D), and trastuzumab (Figure 4E) for 48 hours. Compared with ADCs alone, the combined sequential therapy with TKIs and ADCs inhibited the proliferation of ZR-75-1 cells (Figure 4, B–E, and Supplemental Figure 6E). MFM-223 cells had a similar response as ZR-75-1 cells. Although when compared with trastuzumab or T-DM1 alone, the combined sequential therapy significantly inhibited the cell growth ability of ZR-75-1 or MFM-223 cells, the IC_50_ values were at least 1,000 μg/mL. Only sequential treatment with 10 μg/mL DS-8201 and lapatinib or tucatinib resulted in better inhibitory effects on cell proliferation in ZR-75-1 (Figure 4F, top) and MFM-223 (Figure 4F, bottom) cells. We then performed Western blotting to analyze the expression of c-Myc, Bcl-2, and Bcl-xl in MFM-223 cells. Compared with DS-8201 monotherapy, sequential treatment with DS-8201 and lapatinib or tucatinib could block c-Myc, Bcl-2, and Bcl-xl expression in the MFM-223 cell line (Supplemental Figure 6F).

These results hinted that sequential treatment with DS-8201 and lapatinib or tucatinib could exert antitumor effects in MFM-223 cells by inhibiting cell proliferation and promoting cell apoptosis. Pretreatment with lapatinib or tucatinib followed by sequential DS-8201 treatment could be a potential therapeutic strategy for the TKR subgroup of HER2-low TNBC cells.

To further explore the drug response in patients with HER2-low TKR tumors, we constructed mini patient-derived xenograft (miniPDX) models, as reported previously (33–35), then measured the response for different drugs as well as treatment strategies (Figure 4G). There were 8 patients with the TKR subgroup of HER2-low TNBC and 3 patients with HR^–^ HER2-0 tumors. In line with the results we have reported here, although TKR tumors might respond to DS-8201 (without TKI pretreatment) and T-DM1 (with lapatinib pretreatment) with antiproliferation rates of 30% and 20%, respectively, TKR tumors had higher sensitivity to DS-8201 after lapatinib pretreatment, with an antiproliferation rate as high as 60% (Figure 4H). Together, the results from our TKR cell lines and in vivo animal models strongly suggest that TKR subgroups are potentially targetable, and pretreatment with a TKI (lapatinib) would increase the sensitivity to DS-8201.

### HRD signature in the BSL subgroup and clinical relevance.

**Figure 5:**
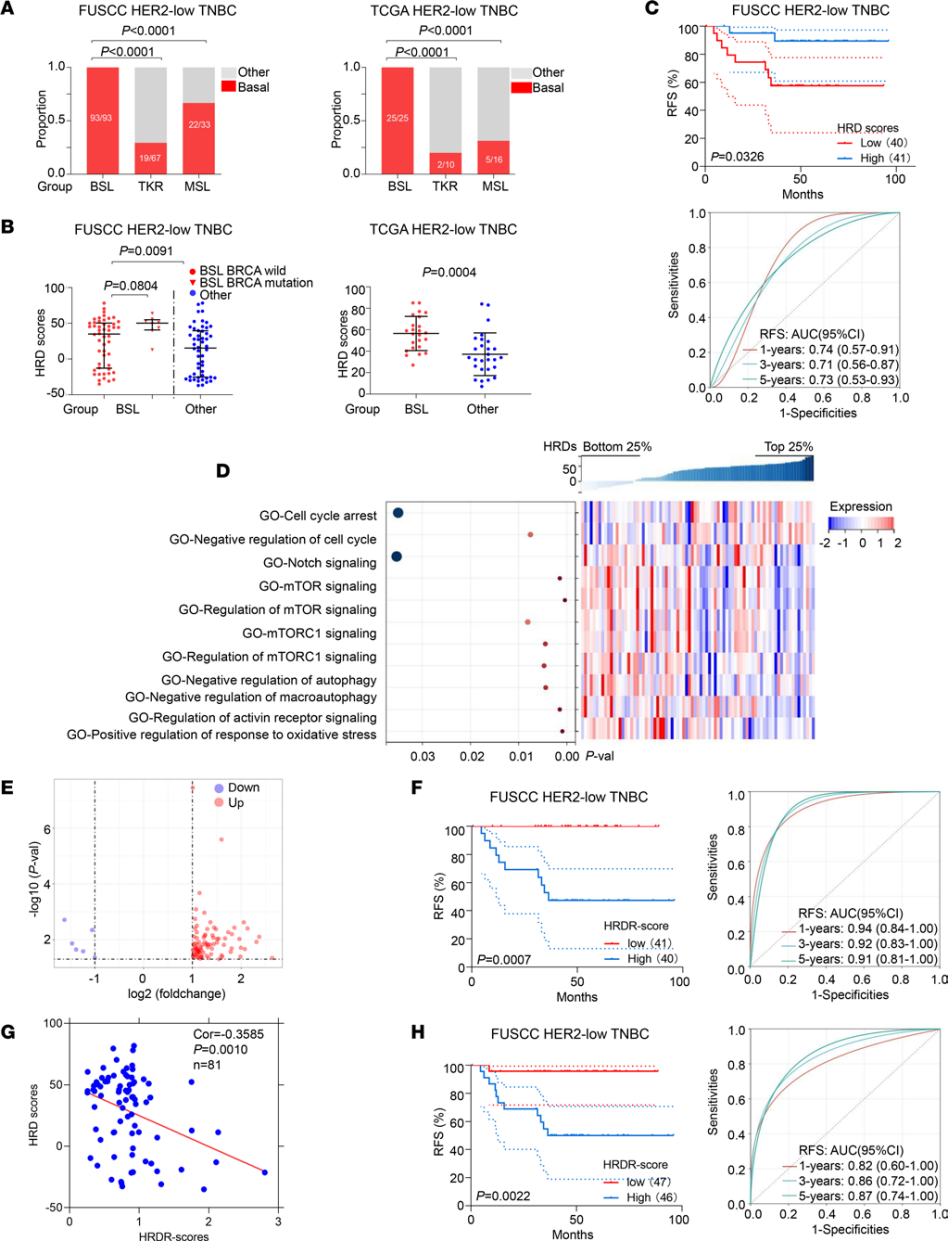
Construction of the HRD signature in the BSL subgroup to predict prognosis. (**A**) Distribution of intrinsic BSL subtypes among the BSL, TKR, and MSL subgroups of HER2-low TNBC from the FUSCC (left) and TCGA (right) data sets (χ^2^ test and Fisher’s exact test). (**B**) Distribution of HRD scores in BSL and other subgroups with BRCA mutation status from the FUSCC data set (left; Mann-Whitney test); distribution of HRD scores in BSL and other subgroups from the TCGA data set (right; Student’s *t* test). (**C**) RFS of BSL patients in the high-HRD versus low-HRD groups (top; log-rank test); AUC of time-dependent ROC analysis (bottom). (**D**) ssGSEA of the BSL subgroup of HER2-low TNBC based on GO data sets are shown in the heatmap. (**E**) Volcano plot illustrating DEGs between the bottom 25% and top 25% of HRD scores in BSL. (**F**) RFS of BSL patients (with HRD scores) with high HRDR score versus low HRDR score groups (left; log-rank test); AUC of time-dependent ROC analysis (right). (**G**) Correlational analysis was performed between HRDR scores and HRD scores in BSL based on the FUSCC data set. (**H**) RFS of total BSL patients (with and without HRD scores) with high HRDR score versus low HRDR score groups (left; log-rank test); AUC of time-dependent ROC analysis. Statistical significance was set at *P* < 0.05. Val, value.

We compared the proportion of the intrinsic BSL subtype among the BSL, TKR, and MSL subgroups and found that the BSL subgroup had the highest probability of intrinsic BSL subtype in both the FUSCC and TCGA data sets (Figure 5A). HRD represents genomic instability and has been identified as an efficient therapeutic biomarker for TNBC (36). The BSL subgroup constituted 57% of the mutation HRD subtype in HER2-low TNBC samples (Figure 2G, right). As illustrated in Figure 5B, the BSL subgroup had significantly higher HRD scores than the other subgroups (FUSCC, *P* = 0.0091; TCGA, *P* = 0.0004). The median HRD score was adopted to classify HER2-low TNBC samples into 2 groups (HRD-high and HRD-low) on the basis of the FUSCC data set. The patients in the HRD-high group had a prominent survival benefit (log-rank *P* = 0.0326; Figure 5C, top). As demonstrated in Figure 5C (bottom), the AUCs of the time-dependent ROC analysis based on HRD scores from the BSL subgroup were 0.74, 0.71, and 0.73 at 1-, 3-, and 5-year relapse-free survival (RFS), respectively.

Then, we analyzed the differentially activated pathways based on the GO data set between the bottom 25% and the top 25% of HRD scores from the patients in the BSL subgroup. The results illustrated that the mTOR and mTORC1 signaling pathways were activated in the bottom 25% of HRD scores (Figure 5D). GSEA verified that the mTOR (NES = 1.6587; *P* = 0.0156) and mTORC1 (NES = 1.5997; *P* = 0.0320) signaling pathways were also activated in the HRD-low BSL subgroup (Supplemental Figure 7A). We further compared mTOR signaling (Supplemental Figure 7B, top) and mTORC1 signaling (Supplemental Figure 7B, bottom) ssGSEA scores among the HRD-low subgroup, the HRD-high BSL subgroup, and other subgroups, and found that mTORC1 signaling ssGSEA scores were higher in the HRD-low BSL subgroup than in the HRD-high BSL and other subgroups. Highly expressed genes in the HRD-low BSL subgroup from the positive regulation mTOR and mTORC1 signaling pathways are shown in Supplemental Figure 7C. These results indicated that the mTOR signaling pathway and mTORC1 could be new potential therapeutic targets for the BSL subgroup in patients with HER2-low TNBC.

Although the HRD score is an excellent prognostic indicator for the BSL subgroup, the methods for detecting HRD are complex. We tried to construct simple and effective strategies to substitute for HRD scores. First, we identified 92 HRD-related DEGs (up: *n* = 86; down: *n* = 6) between the bottom 25% and top 25% of HRD scores in the BSL subgroup (Figure 5E). Then, we integrated RFS time, RFS status, and HRD-related DEG expression and used the Lasso-Cox method for regression analysis to select 5 key genes (*SMCO2*, *C19orf33*, *PAPPA2*, *KCNT1*, and *GABBR2*) at λ = 0.08 and constructed HRD-risk (HRDR) scores (Supplemental Figure 7D). The low-HRDR-score group had a prognostic benefit (log-rank *P* = 0.0007; Figure 5F, left). The AUCs of the time-dependent ROC analysis based on the HRDR scores in the BSL subgroup were 0.94, 0.92, and 0.91 for 1-, 3-, and 5-year RFS, respectively (Figure 5F, right). The correlation analysis based on the HRDR and HRD scores (correlation = –0.3585; *P* = 0.0010; Figure 5G) verified that the HRDR score was a reliable model to replace the HRD score.

To further verify the prognostic role of the HRDR score, we analyzed the prognosis based on the HRDR scores in the BSL subgroup samples (with or without HRD scores). We found that the patients in the high-HRDR-score group had worse RFS (log-rank *P* = 0.0022; Figure 5H, left). The AUCs of the time-dependent ROC analysis based on the HRDR scores in the BSL subgroup were 0.82, 0.86, and 0.87 for 1-, 3-, and 5-year RFS, respectively (Figure 5H, right). These results showed that the HRDR score is a better prognostic indicator than HRD score.

### Upregulated NF-κB signaling pathway in the MSL subgroup and treatment relevance.

**Figure 6:**
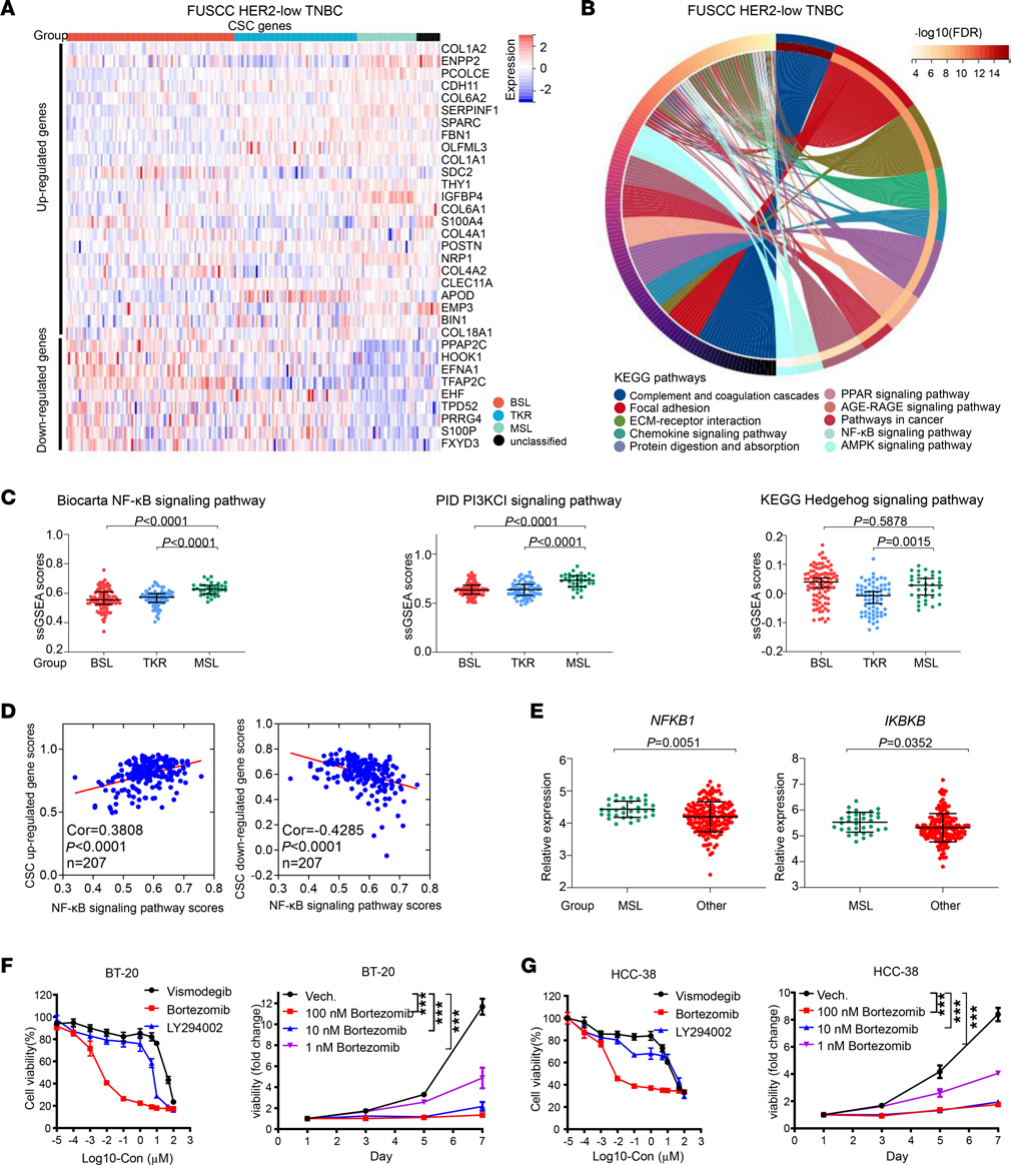
Upregulated NF-κB signaling pathway in the MSL subgroup and treatment relevance. (**A**) Expression of breast CSC genes in HER2-low TNBC. (**B**) Chord plot depicting the relationship between DEGs in MSL subgroups and signaling pathways from the KEGG data set (hypergeometric test). (**C**) ssGSEA scores of the Biocarta NF-κB (left), PID PI3KCI (middle), and KEGG Hedgehog (right) signaling pathways among the BSL, TKR, and MSL subgroups (Kruskal-Wallis test followed by Dunn’s multiple comparisons test). (**D**) Spearman’s correlational analysis was performed between NF-κB signaling pathway ssGSEA scores and CSC upregulated (left), downregulated (right) gene scores in HER2-low TNBC. (**E**) Expression of *NFKB1* (left) and *IKBKB* (right) between MSL and other subgroups (Mann-Whitney test). (**F** and **G**) The CCK-8 assay was used to measure in vitro cell viability of BT-20 (**F**; left) and HCC-38 (**G**; left) cells, which were treated with serial concentrations of CSC-related inhibitors (vismodegib, bortezomib, and LY294002) for 48 hours; after 48 hours, they were incubated with bortezomib (0 nM, 1 nM, 10 nM, and 100 nM). CCK-8 assay results for BT-20 (**F**; right) and HCC-38 (**G**; right) cells. Each point represents the mean and SD (*n* = 6). Statistical significance was set at *P* < 0.05. ***P < 0.001. Cor, correlation; ECM, extracellular matrix.

Gene expression (Figure 6A) and ssGSEA (Figure 1I and Supplemental Figure 1B) suggested that the MSL subgroup might exhibit features of breast cancer stem cells (CSCs). KEGG pathway analysis was performed on the basis of the FUSCC data set, and we found that focal adhesion, extracellular matrix–receptor interaction, and the PPAR, AGE-RAGE, AMPK, and NF-κB signaling pathways could be potential pathways (Figure 6B).

The Hedgehog, NF-κB, and PI3K signaling pathways are common in CSCs (37–39). We estimated the ssGSEA scores of the NF-κB, PI3KCI, and Hedgehog signaling pathways in the HER2-low TNBC samples on the basis of the FUSCC data set and found that the NF-κB signaling pathway and PI3KCI signaling pathway ssGSEA scores were higher in the MSL subgroup (Figure 6C). Correlation analysis among these 3 signaling pathways and the CSC upregulated or downregulated gene scores is shown in Figure 6D and Supplemental Figure 8, A and B. Both the NF-κB signaling pathway and PI3KCI signaling pathway ssGSEA scores were positively correlated with the CSC upregulated gene score and negatively correlated with the CSC downregulated gene score (Figure 6D and Supplemental Figure 8A). TCGA data set was used for external verification, and only the gene set of the NF-κB signaling pathway was enriched in the MSL subgroup (Supplemental Figure 8C).

The expression of critical genes in the NF-κB signaling pathway among the HER2-low TNBC subgroups on the basis of the FUSCC data set is shown in Supplemental Figure 8D. The results demonstrated that the NF-κB signaling pathway activation genes *IKBKB* (40) and *NFKB1* were highly expressed in the MSL subgroup (Figure 6E). These results revealed that the NF-κB signaling pathway could be a potential therapeutic target in the MSL subgroup.

We constructed MSL scores on the basis of expression of *COL1A1*, *COL1A2*, *FBN1*, *NRP1*, *OLFML3*, *PCOLCE*, *SERPINF1*, and *THY1*. MSL scores were higher in the MSL subgroup (*P* < 0.0001; Supplemental Figure 8E, top), and the AUC of MSL scores to estimate the MSL subgroup from HER2-low TNBC samples was 0.82 (Supplemental Figure 8E, bottom), based on FUSCC data set. As external validation data, the results from TCGA data set proved using MSL scores was a reliable prediction method (Supplemental Figure 8F). Then, we selected BT-20 and HCC-38 as the MSL subgroup HER2-low TNBC cells, on the basis of the MSL scores, by analyzing the Genomics of Drug Sensitivity in Cancer database (Supplemental Figure 8G). On the basis of the potential activated pathway in the MSL subgroup, we treated BT-20 and HCC-38 cells with CSC-related inhibitors such as hedgehog inhibitor vismodegib (41), NF-κB pathway inhibitor bortezomib (42), and PI3K inhibitor LY294002 (43).

The results showed that the IC_50_ value of the NF-κB pathway inhibitor bortezomib was appropriate, and the IC_50_ values of the other 2 inhibitors were too high (Figure 6F, left, and Figure 6G, left). The CCK-8 assay demonstrated that bortezomib significantly inhibited the proliferation of BT-20 and HCC-38 cells even at a concentration of 1 ng/mL (Figure 6F, right, and Figure 6G, right). These results verified that the NF-κB pathway could be a potential and reliable target for the therapy of MSL subgroup HER2-low TNBC cells.

## Discussion

The prognosis of HR^–^ and HER2^–^ breast cancer (also known as TNBC) is poor because of limited treatment (11, 12). Recently, the concept of HER2-low TNBC (i.e., low [IHC 1+] or moderate expression [IHC 2+, ISH negative]) has been proposed (9). Although HER2-low and HER2-0 were combined into the HER2^–^ category, compared with HER2-0 tumors, HER2-low tumors have differences in biological characteristics, response to therapy, and prognosis, according to results of 4 prospective neoadjuvant clinical trials (10). Therefore, HER2-low TNBC could be defined as HER2-low TNBC, and new therapeutic targets might be uncovered for this TNBC (Supplemental Table 1).

For therapeutic targets and precise treatment, we identified 3 major subgroups (BSL, TKR, and MSL) of HER2-low TNBC on the basis of transcriptional profiles. Then, we revealed the all-around mutational, copy number, and transcriptional features and demonstrated the robustness of the 3 categories. Of note, among patients in the FUSCC data set with HER2-low TNBC, the proportion in the TKR subgroup was higher than that in TCGA data set. The reason for this phenomenon is somewhat complicated and might be associated with different ethnicity, tumor biology, and heterogeneity of patients enrolled. Although the proportion of the TKR subgroup is higher in our cohort compared with TCGA data set, enriched gene sets of relative pathways in the TKR subgroup were similar between our cohort and TCGA data set.

The luminal androgen receptor (LAR) subgroup, which presented as overexpressed AR and downstream AR targets and coactivators (44), was considered an integral component of TNBC in the Lehmann et al. study (16) andour previous study (13). However, the gene set of the AR signaling pathway was not enriched in the TKR subgroup compared with other HER2-low TNBC. The TKR subgroup could not be regarded simply as the LAR subgroup, at least in HER2-low TNBC.

In our study, the TKR subgroup was identified by activated receptor tyrosine kinase activity. HER2 receptor tyrosine kinase (21) is encoded by *ERBB2*, which is a member of the *ERBB* family, including *ERBB1* (EGFR/HER1) (45), *ERBB3* (HER3) (46), and *ERBB4* (HER4) (47). The *ERBB2* signaling pathway was activated in the TKR subgroup. In addition, activation of positive regulation of protein tyrosine kinase activity and inactivation of negative regulation of protein tyrosine kinase activity were shown in the TKR subgroup. *EGF*, a member of the positive regulation of protein tyrosine kinase activity, was identified as a stimulator of cancer cells (48). In the TKR subgroup, *EGF* mRNA expression was higher than that in the BSL and MSL subgroups, which stemmed from the low copy number loss. We also found that the TKR subgroup was present in HR^+^ and HER2-low TNBC samples, and the MAPK signaling pathway (a classic signaling pathway downstream of HER2) could be activated in HER2-low TNBC regardless of HR status.

We did find that lapatinib and tucatinib induced the accumulation of total HER2, whereas pyrotinib and neratinib deceased the expression of HER2, although all 4 TKIs inhibited the activation of the MAPK pathway (HER2 downstream) in HER2-low TNBC cell lines. As for the opposite effect on HER2 expression, we consider that lapatinib is proven to have a higher affinity for HER2 monomers than it does for EGFR monomers (29), although it is identified as a reversible inhibitor of EGFR/HER2. Similarly, tucatinib is a selective and reversible inhibitor of HER2 (approximately 500-fold selective for HER2 versus EGFR in cell-based assays) (49). Besides, pyrotinib and neratinib are irreversible EGFR/HER2/HER4 inhibitors (50). A sequential combination with DS-8201, a kind of ADC, and lapatinib or tucatinib could be a potential therapy for the TKR subgroup of HER2-low TNBC. Despite the efficacy of DS-8201 in patients with advanced HER2-low TNBC (7, 8), progression-free survival is much shorter in patients with HER2-low cancer than in patients with HER2^+^ disease (51).

For the first time, to our knowledge, we reveal a strategy for sensitizing DS-8201 in HER2-low cells. Regarding the biological mechanism, we speculated that there was a feedback loop in HER2-low samples, paralleling HER2-overexpressing breast cancer (29). In HER-low samples, lapatinib or tucatinib could promote the accumulation of inactive or nonfunctional HER2 receptors by preventing HER2 ubiquitination, enhancing the stability of HER2, and prolonging the half-lives of inactive HER2 receptors, which might facilitate DS-8201 binding and activity to increase DS-8201–mediated, Ab-dependent, cell-mediated cytotoxicity.

HRD, which represents genomic instability (36), can be used to identify patients who might benefit from DNA-damaging agents (52) and predict the response to platinum-neoadjuvant chemotherapy (53). HRD scores were higher in the BSL subgroup and could be a potential method to identify BSL subgroups. New therapeutic targets are needed for BSL subgroup HER2-low TNBC. We found that the mTOR signaling pathway and mTORC1 signaling pathway were activated in the HRD-low group of the BSL subgroup, which had a poor prognosis. mTOR signaling promotes cell proliferation in human tumors (54) on the basis of the availability of growth factors, nutrients, and energy (55). Activation of the mTOR and mTORC1 signaling pathways could promote protein synthesis and cell growth. Our results revealed that the mTOR and mTORC1 signaling pathways might be potential therapeutic targets for HRD-low BSL subgroup HER2-low TNBC.

The MSL subgroup exhibited features of breast CSCs. The Hedgehog, NF-κB, and PI3K signaling pathways are common in CSCs (37–39). We found that the NF-κB signaling pathway was activated in the MSL subgroup of HER2-low TNBC samples. Cytological verification by the NF-κB pathway inhibitor bortezomib (32) suggested that the NF-κB pathway could be a potential and reliable target for the therapy of MSL subgroup HER2-low TNBC cells.

Our study reveals the genomic and transcriptomic landscape of Chinese patients with HER2-low TNBC, and our main findings were successfully validated in TCGA data set. We also provide potential therapeutic targets for each subgroup of HER2-low TNBC, which may be significant when classifying TNBCs. Prospective multicenter studies of patients with HER-low TNBC should be planned to test the clinical relevance of our findings.

There are several limitations in our study. First, we focused more on HR^–^ breast cancer than on HR^+^. Identifying subgroups of HR^–^ and HER2-low tumors (belonging to TNBC) might be more meaningful and clinically relevant, given the limited strategies for treating TNBC. Second, the sample size was relatively small and requires further external replication. Third, the lack of proteomics and metabolomics cooperation in this multiomics era leads to insufficient insights into HER2-low subgroups. Fourth, although animal models and clinical trials are critical and essential for translating the potential therapeutic targets of each subgroup into clinical practice, the current research did not provide such information. And fifth, we realized that IHC HER2 status plays a crucial role in subgrouping HER2-low TNBC, and further research on biomarker-based approaches to classify HER2-low subgroups is necessary and proper.

In summary, we performed this multiomics study of HR^–^ and HER2-low TNBC and identified BSL, TKR, and MSL subgroups, which had distinct features and are potential therapeutic targets. It was noteworthy that the TKR subgroup, which was present in HR^+^ and HR^–^ samples, had activated HER2/MAPK signaling. Pretreatment of HER2-low TKR breast cancer cells with lapatinib or tucatinib inhibited HER2 signaling and induced increased expression of nonfunctional HER2, which resulted in the sensitization of sequential use of DS-8201. Our findings identify clinically relevant subgroups and provide potential therapeutic strategies for the previously targetless HER2-low TNBC subtype.

## Methods

### Data set collection, processing, and analysis.

For details, see Supplemental Methods.

### Cell lines and cell culture.

The human breast cancer cell lines MDA-MB-231, SK-BR-3, ZR-75-1, and MFM-223 were purchased from ATCC. MDA-MB-231 and ZR-75-1 breast cancer cells were cultured in RPMI 1640 medium supplemented with 5% FBS (Biological Industries) and 1% penicillin and streptomycin antibiotic (Beyotime), and SK-BR-3 and MFM-223 cells were cultured in DMEM supplemented with 10% FBS and 1% penicillin and streptomycin antibiotic. All of the cell lines were tested and authenticated. All cells were cultured at 37°C in a humidified atmosphere containing 5% CO_2_. The cell lines were mycoplasma-free and authenticated by PCR analysis monthly.

### Cell proliferation assay.

The CCK-8 colorimetric assay (Dojindo Laboratories) measured cell proliferation and viability with 3 replicates. MDA-MB-231, SK-BR-3, ZR-75-1, and MFM-223 cells were seeded in 96-well plates with 5 × 10^3^ cells/well and treated with serially diluted concentrations of TKIs (lapatinib, pyrotinib, neratinib, and tucatinib) at 24 hours. After culture for about 48 hours, cell viability was assessed using the CCK-8 kit and measured at 450 nm with a microplate reader.

The following treatment combinations were administered: ZR-75-1 cells were treated with lapatinib (1 μM), tucatinib (1 μM), or vehicle control (Vech.) for 48 hours and sequentially treated with DS-8201 for 48 hours; MFM-223 cells were treated with lapatinib (10 μM), tucatinib (1 μM), or Vech. for 48 hours and sequentially with DS-8201 for 48 hours; ZR-75-1 cells were treated with lapatinib (1 μM), tucatinib (1 μM), or Vech. for 48 hours and sequentially with T-DM1 for 48 hours; MFM-223 cells were treated with lapatinib (10 μM), tucatinib (1 μM), or Vech. for 48 hours and sequentially with T-DM1 for 48 hours; ZR-75-1 cells were treated with lapatinib (1 μM), tucatinib (1 μM), or Vech. for 48 hours and sequentially with trastuzumab for 48 hours; and MFM-223 cells were treated with lapatinib (10 μM), tucatinib (1 μM), or Vech. for 48 hours and sequentially with trastuzumab for 48 hours. Then cell viability was assessed using the CCK-8 kit and measured at 450 nm with a microplate reader.

ZR-75-1 cells were incubated with lapatinib (1 μM), tucatinib (1 μM), or Vech. for 48 hours and then with DS-8201 (10 μg/mL) or Vech. For 48 hours, MFM-223 cells were incubated with lapatinib (10 μM), tucatinib (1 μM), or Vech. for 48 hours and then with DS-8201 (10 μg/mL) for 48 hours. After 48 hours of incubation with lapatinib, tucatinib (1 μM), or Vech for 48 hours, cell viability was estimated every 48 hours (days 1 to 7).

BT-20 and HCC-38 cells were seeded in 96-well plates with 5 × 10^3^ cells/well and treated with serially diluted concentrations of CSC-related inhibitors (vismodegib, bortezomib, and LY294002) at 24 hours. After culture for about 48 hours, cell viability was assessed using the CCK-8 kit and measured at 450 nm with a microplate reader.

BT-20 and HCC-38 cells were incubated with bortezomib (0 nM, 1 nM, 10 nM, and 100 nM) for 48 hours, and then cell viability was assessed every 48 hours (days 1 to 7).

### Western blotting.

Western blotting was performed as described previously (56). All experiments were repeated more than 3 times. Ab information is provided in Supplemental Methods.

### MiniPDX model.

We conducted the in vivo pharmacological tests using the OncoVee miniPDX assay (LIDE Biotech) as previously described (33–35). In brief, fresh surgical breast cancer specimens (*n* = 8 from the TKR subgroup of HER2-low TNBC and *n* = 3 from the HR^–^ HER2-0 [pure TNBC]) were acquired from women (average age, 46 years) at FUSCC. Specimens were then washed with HBSS to remove necrotic tumor tissues and nontumor tissues. A fraction of tissue was retained for RNA extraction. The rest was fragmented and digested with collagenase at 37°C for 1–2 hours. Cells were collected, followed by the removal of blood cells and fibroblasts, and then were suspended to fill the HBSS-washed capsules. Each capsule contained approximately 2,000 cells, and capsules derived from the same specimen were assigned to the baseline, control (saline), and different treatment groups. Capsules were implanted s.c. into 6-week-old, female BALB/c nude mice.

One day after inoculation of tumor cells, the tumor-bearing mice were given the following drugs for 7 days: for the no-pretreatment group, there was no treatment in the first 3 days, then 1 dose each of T-DM1 10 mg/kg, i.v. and DS-8201 10 mg/kg, i.v. was administered; for the pretreatment group, lapatinib 30 mg/kg was orally administered twice a day for the first 3 days, then 1 dose each of T-DM1 10 mg/kg and DS-8201 4 mg/kg was given i.v. Each treatment (control or different strategies) was performed in triplicate capsules. Finally, the antitumor activity was evaluated on the basis of RLUs using the CellTiter-Glo Luminescent Cell Viability Assay (Promega). The proliferation rate was calculated using the following formula: relative viability = (RLU of treatment at D7 – RLU of baseline)/(RLU of control at D7 – RLU of baseline) × 100. The study flowchart of miniPDX is shown in Figure 4G.

### Statistics.

We used SPSS Statistics 22 (IBM), GraphPad Prism 8.0 (GraphPad Software), and R software (version 4.0.4; http://www.r-project.org) to calculate and analyze data and construct graphs. We performed Student’s *t* test to compare continuous variables of 2 groups and the Mann-Whitney test to compare ordered categorical variables. One-way or 2-way ANOVA was used for comparing continuous variables among multiple comparisons (Dunnett’s *t* test as a follow-up test), and the Kruskal-Wallis test was used for ordered categorical variables. Pearson’s χ^2^ test and Fisher’s exact test were used to analyze unordered categorical variables according to specific circumstances. A log-rank test was used to construct survival curves. Statistical significance was set at the level of 2-tailed *P* < 0.05.

### Study approval.

The use of clinical cancer samples was approved by the Ethics Committee of FUSCC (Protocol number: 050432 4 2108) and each patient signed an informed-consent document. The animal model protocol was approved by the IACUC (number: FUSCC IACUC S2022 0245).

### Data availability.

All data can be accessed in the National Encyclopedia of Omics Data at http://www.biosino.org/node/project/detail/OEP000155 Microarray data and sequence data have also been deposited in the National Center for Biotechnology Information’s Gene Expression Omnibus (GEO) (OncoScan Array; GEO: GSE118527) and the Sequence Read Archive (SRA) (RNA-Seq and WES; SRA: SRP157974). Individual values for all other data are available in the Supporting Data Values XLS file.

## Author contributions

KDY outlined the manuscript. LC, CCL, SYZ, JYG, YFC, and DM contributed to literature review, data collection, data analysis, and manuscript proofreading. CCL and LC performed cell experiments and drafted the manuscript. SYZ, JYG, YFC, and DM edited the manuscript. ZMS assisted in guiding the experiment. All authors approved the final manuscript.

## Supplementary Material

Supplemental data

Supporting data values

## Version 1 11/22/2023

Electronic publication

## References

[1] Hudis CA (2007). Trastuzumab--mechanism of action and use in clinical practice. N Engl J Med.

[2] Loibl S, Gianni L (2017). HER2-positive breast cancer. Lancet.

[3] Wolff AC (2018). Human epidermal growth factor receptor 2 testing in breast cancer: American Society of Clinical Oncology/College of American Pathologists Clinical Practice Guideline focused update. J Clin Oncol.

[4] Nakada T (2019). The latest research and development into the antibody-drug conjugate, [fam-] trastuzumab deruxtecan (DS-8201a), for HER2 cancer therapy. Chem Pharm Bull (Tokyo).

[5] Banerji U (2019). Trastuzumab duocarmazine in locally advanced and metastatic solid tumours and HER2-expressing breast cancer: a phase 1 dose-escalation and dose-expansion study. Lancet Oncol.

[6] Takegawa N (2019). [fam-] trastuzumab deruxtecan, antitumor activity is dependent on HER2 expression level rather than on HER2 amplification. Int J Cancer.

[7] Modi S (2020). Antitumor activity and safety of trastuzumab deruxtecan in patients with HER2-low-expressing advanced breast cancer: results from a phase Ib study. J Clin Oncol.

[8] Modi S (2022). Trastuzumab deruxtecan in previously treated HER2-low advanced breast cancer. N Engl J Med.

[9] Schalper KA (2014). A retrospective population-based comparison of HER2 immunohistochemistry and fluorescence in situ hybridization in breast carcinomas: impact of 2007 American Society of Clinical Oncology/College of American Pathologists criteria. Arch Pathol Lab Med.

[10] Denkert C (2021). Clinical and molecular characteristics of HER2-low-positive breast cancer: pooled analysis of individual patient data from four prospective, neoadjuvant clinical trials. Lancet Oncol.

[11] Dent R (2007). Triple-negative breast cancer: clinical features and patterns of recurrence. Clin Cancer Res.

[12] Venkitaraman R (2010). Triple-negative/basal-like breast cancer: clinical, pathologic and molecular features. Expert Rev Anticancer Ther.

[13] Jiang YZ (2019). Genomic and transcriptomic landscape of triple-negative breast cancers: subtypes and treatment strategies. Cancer Cell.

[14] Newman AM (2015). Robust enumeration of cell subsets from tissue expression profiles. Nat Methods.

[15] Angelova M (2015). Characterization of the immunophenotypes and antigenomes of colorectal cancers reveals distinct tumor escape mechanisms and novel targets for immunotherapy. Genome Biol.

[16] Lehmann BD (2016). Refinement of triple-negative breast cancer molecular subtypes: implications for neoadjuvant chemotherapy selection. PLoS One.

[17] Ring BZ (2016). Generation of an algorithm based on minimal gene sets to clinically subtype triple negative breast cancer patients. BMC Cancer.

[18] Topalian SL (2012). Safety, activity, and immune correlates of anti-PD-1 antibody in cancer. N Engl J Med.

[19] Robert C (2014). Anti-programmed-death-receptor-1 treatment with pembrolizumab in ipilimumab-refractory advanced melanoma: a randomised dose-comparison cohort of a phase 1 trial. Lancet.

[20] Warren CM, Landgraf R (2006). Signaling through ERBB receptors: multiple layers of diversity and control. Cell Signal.

[21] Strickler JH (2022). Diagnosis and treatment of ERBB2-positive metastatic colorectal cancer: a review. JAMA Oncol.

[22] Tarantino P (2022). Aiming at a tailored cure for ERBB2-positive metastatic breast cancer: a review. JAMA Oncol.

[23] Lavoie H (2018). MEK drives BRAF activation through allosteric control of KSR proteins. Nature.

[24] Keshet Y, Seger R (2010). The MAP kinase signaling cascades: a system of hundreds of components regulates a diverse array of physiological functions. Methods Mol Biol.

[25] Saura C (2020). Neratinib plus capecitabine versus lapatinib plus capecitabine in HER2-positive metastatic breast cancer previously treated with ≥ 2 HER2-directed regimens: phase III NALA trial. J Clin Oncol.

[26] Xu B (2021). Pyrotinib plus capecitabine versus lapatinib plus capecitabine for the treatment of HER2-positive metastatic breast cancer (PHOEBE): a multicentre, open-label, randomised, controlled, phase 3 trial. Lancet Oncol.

[27] Medina PJ, Goodin S (2008). Lapatinib: a dual inhibitor of human epidermal growth factor receptor tyrosine kinases. Clin Ther.

[28] Borges VF (2018). Tucatinib combined with ado-trastuzumab emtansine in advanced ERBB2/HER2-positive metastatic breast cancer: a phase 1b clinical trial. JAMA Oncol.

[29] Scaltriti M (2009). Lapatinib, a HER2 tyrosine kinase inhibitor, induces stabilization and accumulation of HER2 and potentiates trastuzumab-dependent cell cytotoxicity. Oncogene.

[30] Lambert JM, Chari RV (2014). Ado-trastuzumab emtansine (T-DM1): an antibody-drug conjugate (ADC) for HER2-positive breast cancer. J Med Chem.

[31] Ogitani Y (2016). DS-8201a, a novel HER2-targeting ADC with a novel DNA topoisomerase I inhibitor, demonstrates a promising antitumor efficacy with differentiation from T-DM1. Clin Cancer Res.

[32] Ogitani Y (2016). Bystander killing effect of DS-8201a, a novel anti-human epidermal growth factor receptor 2 antibody-drug conjugate, in tumors with human epidermal growth factor receptor 2 heterogeneity. Cancer Sci.

[33] Li C (2020). Integrated omics of metastatic colorectal cancer. Cancer Cell.

[34] Chen YY (2022). Copy number amplification of ENSA promotes the progression of triple-negative breast cancer via cholesterol biosynthesis. Nat Commun.

[35] Zhang F (2018). Characterization of drug responses of mini patient-derived xenografts in mice for predicting cancer patient clinical therapeutic response. Cancer Commun (Lond).

[36] Chopra N (2020). Homologous recombination DNA repair deficiency and PARP inhibition activity in primary triple negative breast cancer. Nat Commun.

[37] Sun Y (2014). Estrogen promotes stemness and invasiveness of ER-positive breast cancer cells through Gli1 activation. Mol Cancer.

[38] Lu H (2014). A breast cancer stem cell niche supported by juxtacrine signalling from monocytes and macrophages. Nat Cell Biol.

[39] Al-Dhfyan A (2017). Aryl hydrocarbon receptor/cytochrome P450 1A1 pathway mediates breast cancer stem cells expansion through PTEN inhibition and β-Catenin and Akt activation. Mol Cancer.

[40] Kondylis V (2015). NEMO prevents steatohepatitis and hepatocellular carcinoma by inhibiting RIPK1 kinase activity-mediated hepatocyte apoptosis. Cancer Cell.

[41] Kundu S (2022). The scaffolding protein DLG5 promotes glioblastoma growth by controlling Sonic Hedgehog signaling in tumor stem cells. Neuro Oncol.

[42] Spevak CC (2020). Hematopoietic stem and progenitor cells exhibit stage-specific translational programs via mTOR- and CDK1-dependent mechanisms. Cell Stem Cell.

[43] Xia P (2019). Lateral inhibition in cell specification mediated by mechanical signals modulating TAZ activity. Cell.

[44] Lehmann BD (2011). Identification of human triple-negative breast cancer subtypes and preclinical models for selection of targeted therapies. J Clin Invest.

[45] Gullick WJ, Srinivasan R (1998). The type 1 growth factor receptor family: new ligands and receptors and their role in breast cancer. Breast Cancer Res Treat.

[46] Carraway KL (1997). Roles of ErbB-3 and ErbB-4 in the physiology and pathology of the mammary gland. J Mammary Gland Biol Neoplasia.

[47] Lucas LM (2022). The yin and yang of ERBB4: tumor suppressor and oncoprotein. Pharmacol Rev.

[48] Shao G (2018). The E3 ubiquitin ligase NEDD4 mediates cell migration signaling of EGFR in lung cancer cells. Mol Cancer.

[49] Moulder SL (2017). Phase I study of ONT-380, a HER2 inhibitor, in patients with HER2^+^-advanced solid tumors, with an expansion cohort in HER2^+^ metastatic breast cancer (MBC). Clin Cancer Res.

[50] Schlam I, Swain SM (2021). HER2-positive breast cancer and tyrosine kinase inhibitors: the time is now. NPJ Breast Cancer.

[51] Modi S (2020). Trastuzumab deruxtecan in previously treated HER2-positive breast cancer. N Engl J Med.

[52] Timms KM (2014). Association of BRCA1/2 defects with genomic scores predictive of DNA damage repair deficiency among breast cancer subtypes. Breast Cancer Res.

[53] Telli ML (2016). Homologous recombination deficiency (HRD) score predicts response to platinum-containing neoadjuvant chemotherapy in patients with triple-negative breast cancer. Clin Cancer Res.

[54] Menon S, Manning BD (2008). Common corruption of the mTOR signaling network in human tumors. Oncogene.

[55] Laplante M, Sabatini DM (2012). mTOR signaling in growth control and disease. Cell.

[56] Liu C (2021). ALDH1A1 activity in tumor-initiating cells remodels myeloid-derived suppressor cells to promote breast cancer progression. Cancer Res.

