# Supplementary material for: Multiomics of HER2-low triple-negative breast cancer identifies a receptor tyrosine kinase–relevant subgroup with therapeutic prospects
Source: JCI Insight. 2023 Nov 22;8(22):e172366. doi: 10.1172/jci.insight.172366 (PMC10721318; doi:10.1172/jci.insight.172366)
Supplement: Supplemental data [file jciinsight-8-172366-s040.pdf]

Figure S1

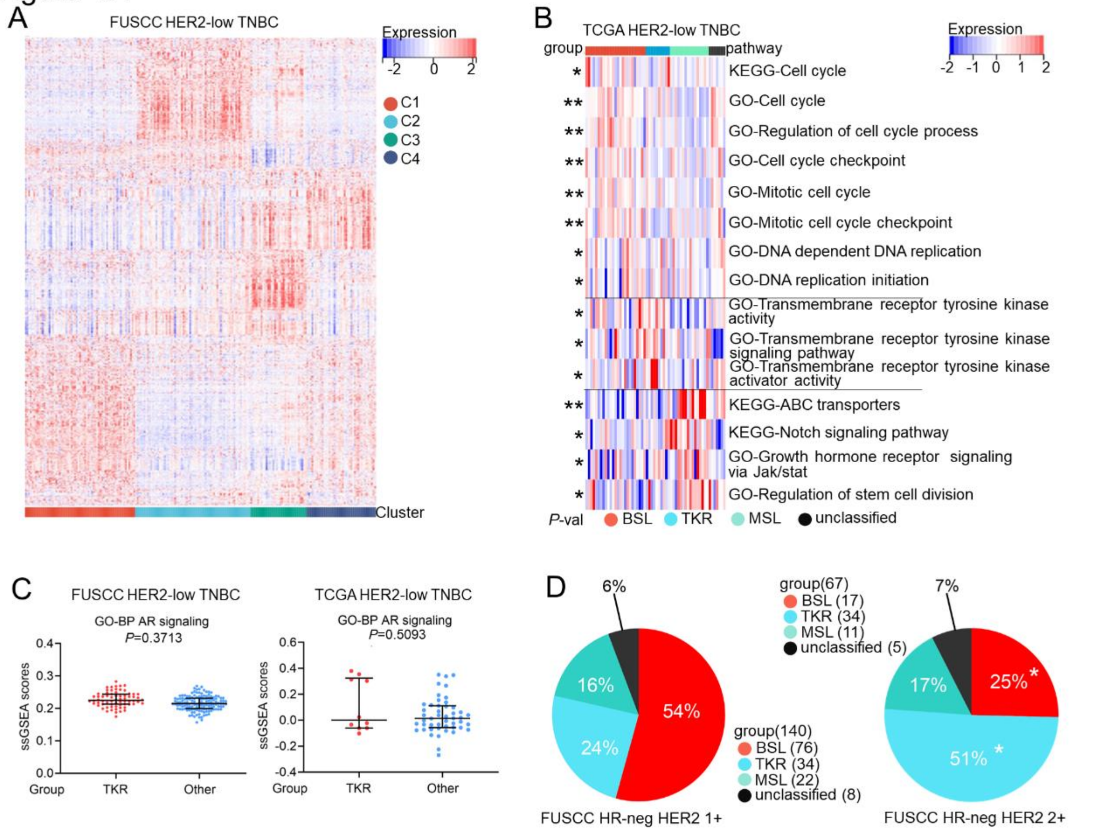

Supplementary Figure 1.

A, Top 2000 most variable mRNAs of 207 HER2-low TNBC were shown in the heat-map. B, ssGSEA of 58 HER2-low TNBC from TCGA dataset based on KEGG and GO datasets were shown in the heat-map. C, Androgen receptor (AR) signaling ssGSEA scores between TKR and other subgroup of HER2-low TNBC from FUSCC (left) and TCGA (right) datasets (Mann-Whitney test). D, Distribution of HR-negative (neg) and HER2 1+ breast cancer mRNA subgroups in FUSCC dataset (left), distribution of HR-neg and HER2 2+ breast cancer mRNA subgroups in FUSCC dataset (right, chi-square test). $P<0.05$ was considered as statistical significance. *, $P<0.05$. **, $P<0.01$.

Figure S2

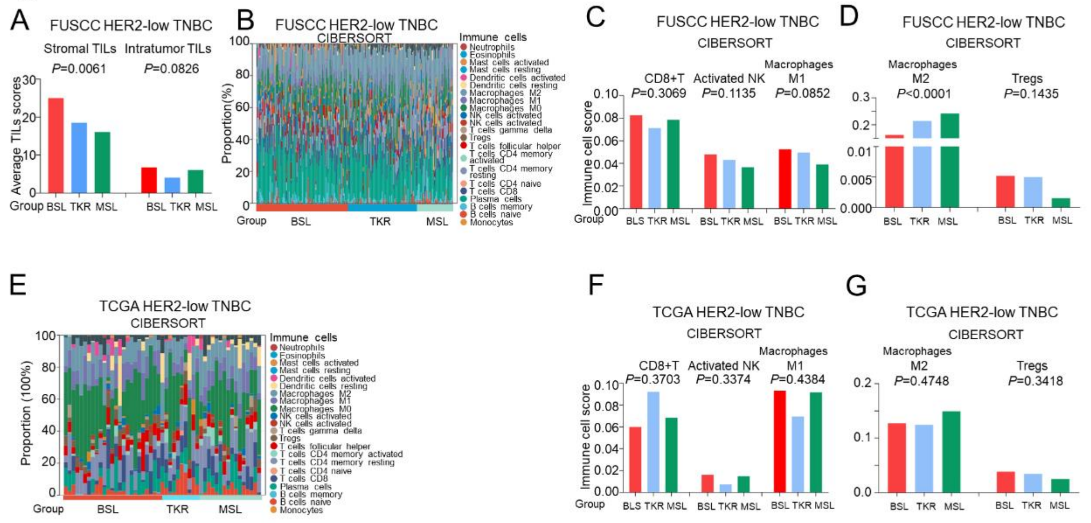

Supplementary Figure 2.

A, Average stromal and intratumoral TIL scores in the BSL, TKR, and MSL subgroups (Kruskal-Wallis test). B, Bar chart illustrating the relative abundance of 22 kinds of immune cells on the basis of CIBERSORT among the BSL, TKR, and MSL subgroups. C and D, Relative abundance of 3 immune-activated cells (C) and 2 immune-inhibited cells (D) in the BSL, TKR, and MSL subgroups on the basis of CIBERSORT (Kruskal-Wallis test). E, Bar chart illustrated the relative abundance of 22 kinds of immune cells based on CIBERSORT among BSL, TKR, and MSL subgroups. F and G, Relative abundance of 3 immune-activated cells (F) and 2 immune-inhibited cells (G) in BSL, TKR, and MSL subgroups based on CIBERSORT (Kruskal-Wallis test). *P*<0.05 was considered as statistical significance.

Figure S3

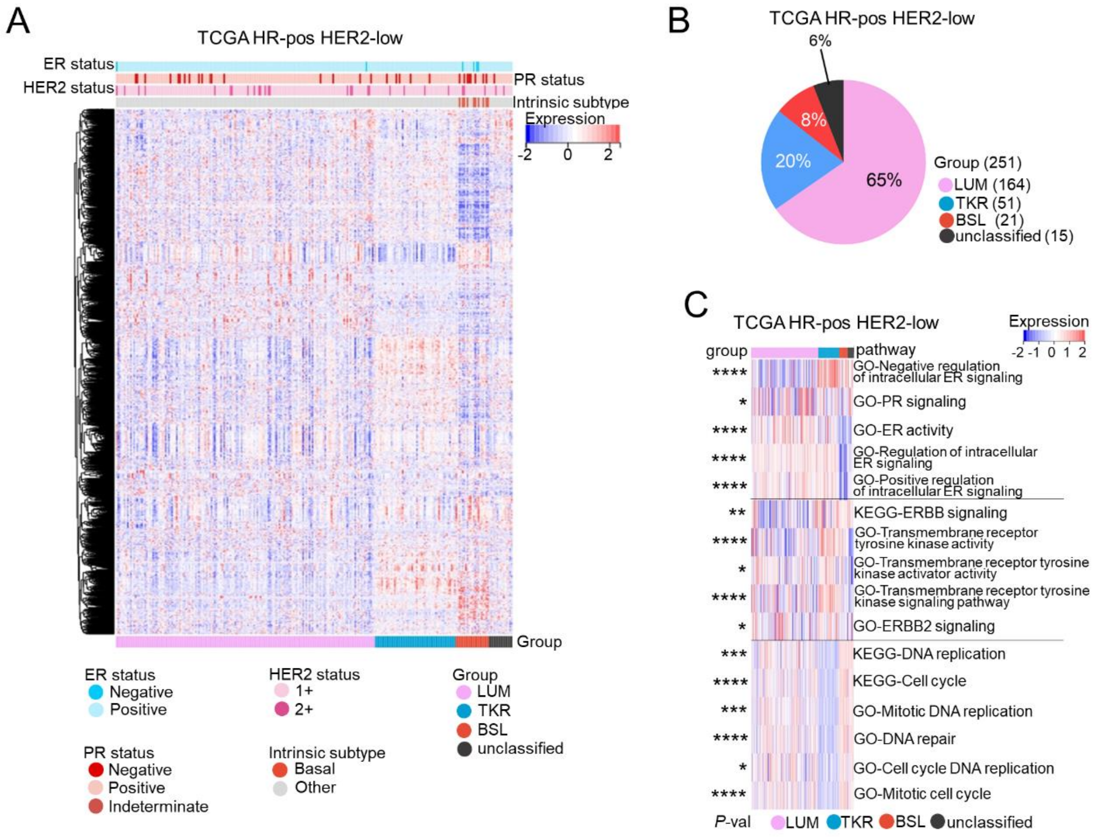

Supplementary Figure 3.

A, Top 2000 most variable mRNAs of HR-positive (pos) and HER2-low breast cancer in the TCGA dataset were shown in the heat-map with clinical and molecular features annotated. B, Distribution of HR-pos and HER2-low breast cancer mRNA subgroups in TCGA dataset. C, ssGSEA of HR-pos and HER2-low breast cancer from TCGA dataset based on KEGG and GO datasets were shown in the heat-map. $P<0.05$ was considered as statistical significance. *, $P<0.05$. **, $P<0.01$. ***, $P<0.001$. ****, $P<0.0001$.

Figure S4

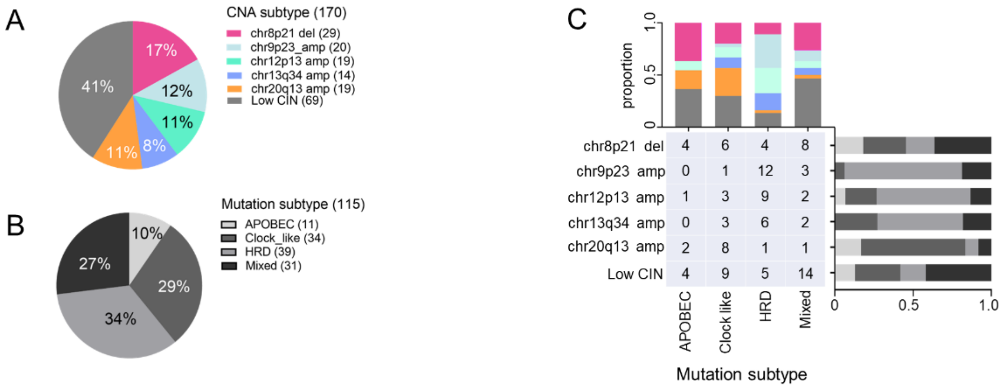

Supplementary Figure 4.

A, Distribution of HER2-low TNBC CNA subtypes. B, Distribution of HER2-low TNBC mutation subtypes. C, Relationships between CNA subtypes and mutation subtypes. A-C were based on HER2-low TNBC breast cancer from FUSCC dataset.

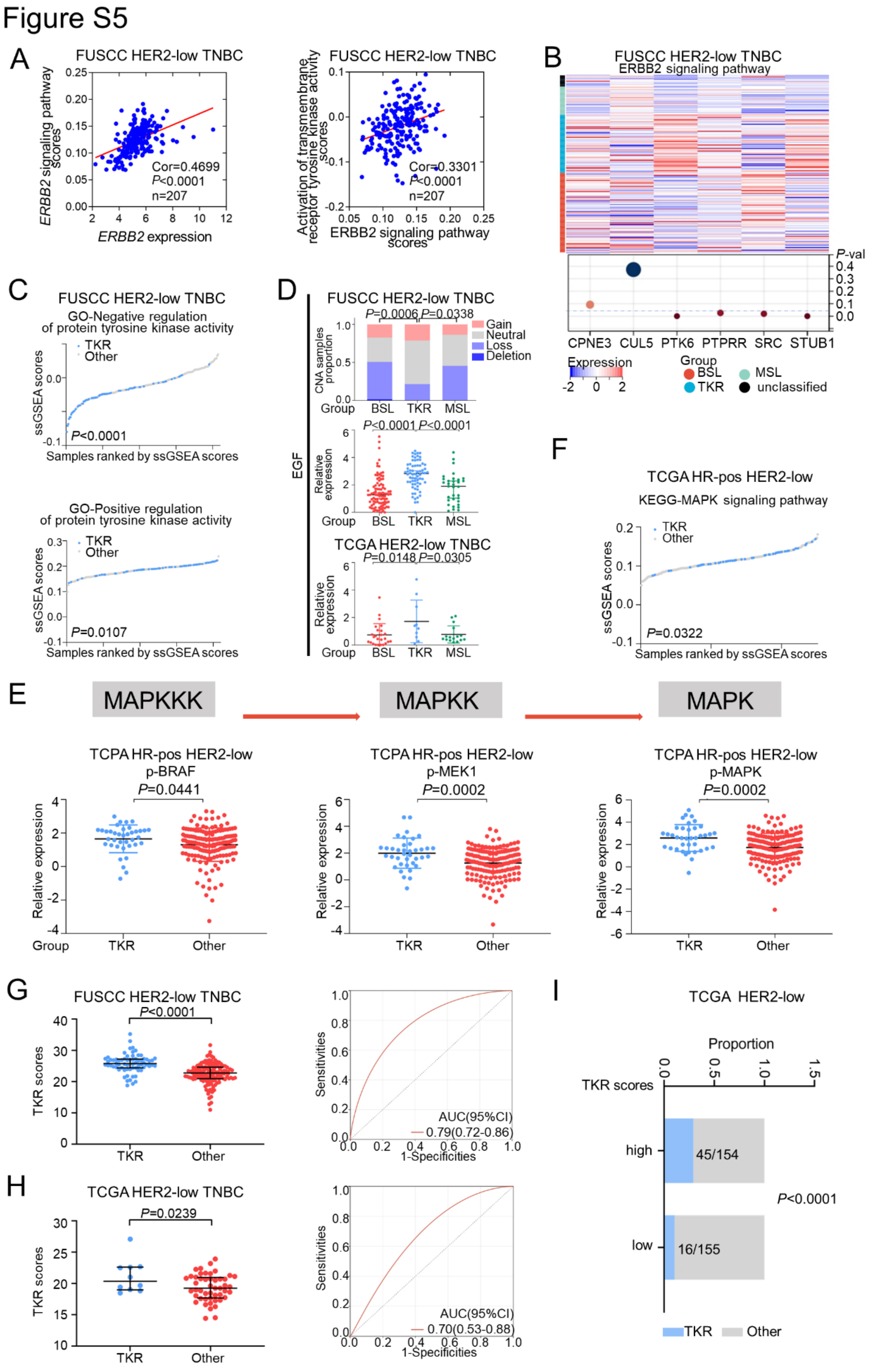

Supplementary Figure 5.

A, Spearman correlational analysis was performed between *ERBB2* expression and *ERBB2* signaling pathway ssGSEA scores (left), *ERBB2* signaling pathway ssGSEA scores and activation of transmembrane receptor tyrosine kinase activity ssGSEA scores (right) in TKR subgroup. B, Expression levels of 6 ERBB*2* signaling pathway-related genes are shown in the heatmap, and *P* values are shown as bubble plots. C, GO-negative regulation of protein tyrosine kinase activity (top) and GO-positive regulation of protein tyrosine kinase activity (bottom) ssGSEA scores were available, student's t test. D, CNA of *EGF* among the BSL, TKR, and MSL subgroups from the FUSCC dataset (top, chi-square test), relative expression of *EGF* among the BSL, TKR, and MSL subgroups from the FUSCC (middle) and TCGA (bottom, Kruskal-Wallis test followed by Dunn's multiple comparisons test) datasets. E, Relative expression of phospho-BRAF (p-BRAF, MAPKKK, left), phospho-MEK1 (p-MEK1, MAPKK, middle), and p-MAPK (right) between TKR and other subgroups from HR-pos and HER2-low breast cancer in TCPA dataset (student's t test). F, MAPK signaling pathway ssGSEA scores between TKR and other subgroups from HR-pos and HER2-low breast cancer in TCGA dataset (student's t test). G and H, TKR scores between TKR and other subgroups (left, Mann-Whitney test) and area under the standard receiver operating characteristic (ROC) curve (AUC) of TKR scores to estimate the TKR subgroup (right) from HER2-low TNBC samples based on FUSCC (G) and TCGA (H) datasets. I, The proportion of TKR subgroup between high and low TKR scores group from total HER2-low breast cancer in TCGA dataset (chi-square test). $P<0.05$ was considered as statistical significance.

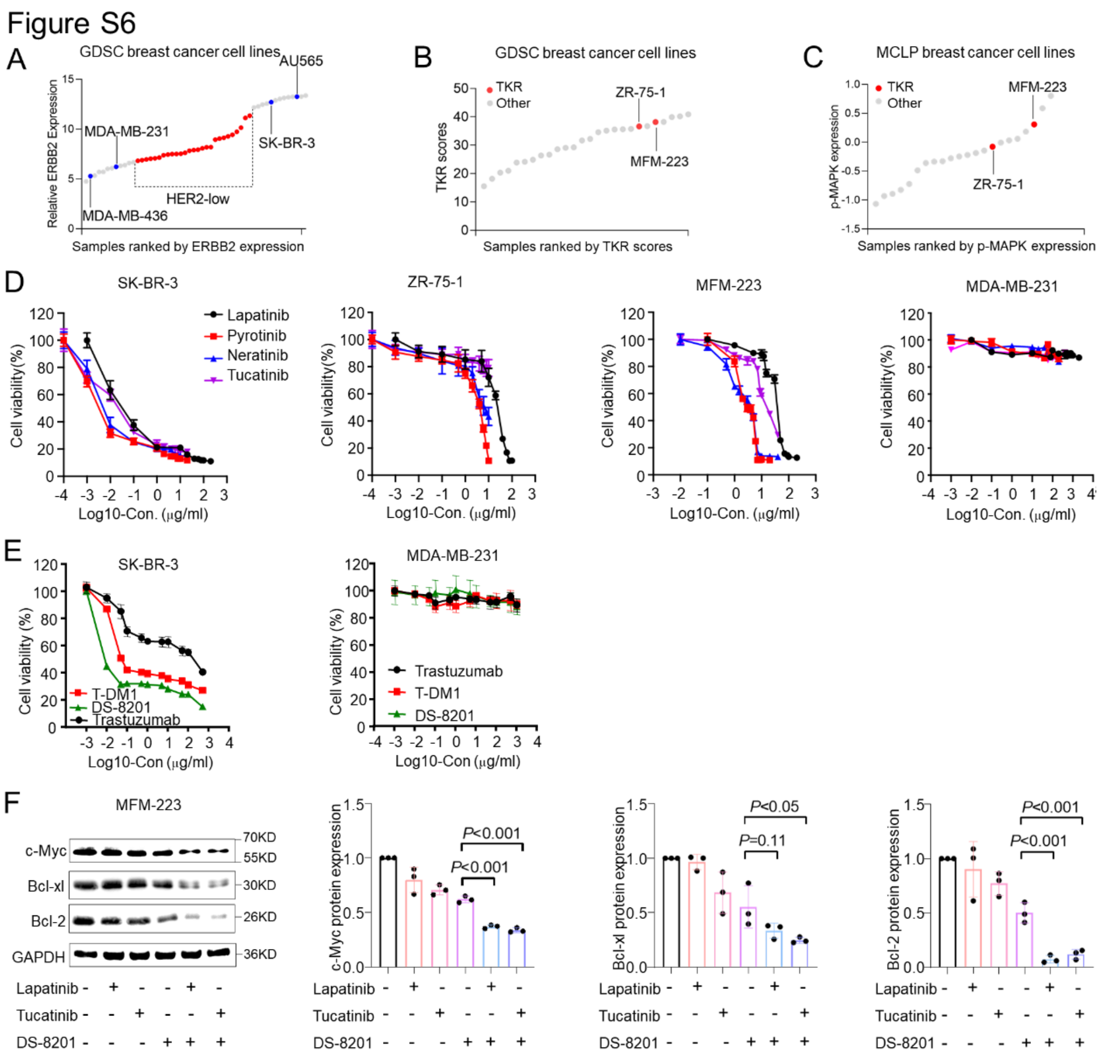

Supplementary Figure 6.

A, *ERBB2* expression in 54 kinds of breast cancer cells was observed, and 27 middle half cells were defined as the HER2-low subtype. B, TKR scores of ZR-75-1, MFM-223, and other HER2-low breast cancers are illustrated. Data in A and B were from the Genomics of Drug Sensitivity in Cancer (GDSC) dataset. C, p-MAPK levels in ZR-75-1, MFM-223, and other HER2-low breast cancers were revealed on the basis of The MD Anderson Cell Lines Project (MCLP) dataset. D, CCK-8 assay measured cell viability of SK-BR-3, ZR-75-1, MFM-223, and MDA-MB-231 cells in vitro. The cells were treated with serial concentrations Lapatinib, Pyrotinib, Neratinib, and Tucatinib

for 48 hours. Each point represented the mean and SD (n =6). E, CCK-8 assay measured the cell viability of SK-BR-3 and MDA-MB-231 cells in vitro. F, Western blot showing c-Myc, Bcl-xl, Bcl-2, and GAPDH expression in MFM-223 cells incubated with Lapatinib (10 μM), Tucatinib (1 μM), or Vech. for 48 hours and then DS-8201 (10 μg/ml) for 48 hours (1-way ANOVA followed by Dunnett-t test). P<0.05 was considered as statistical significance.

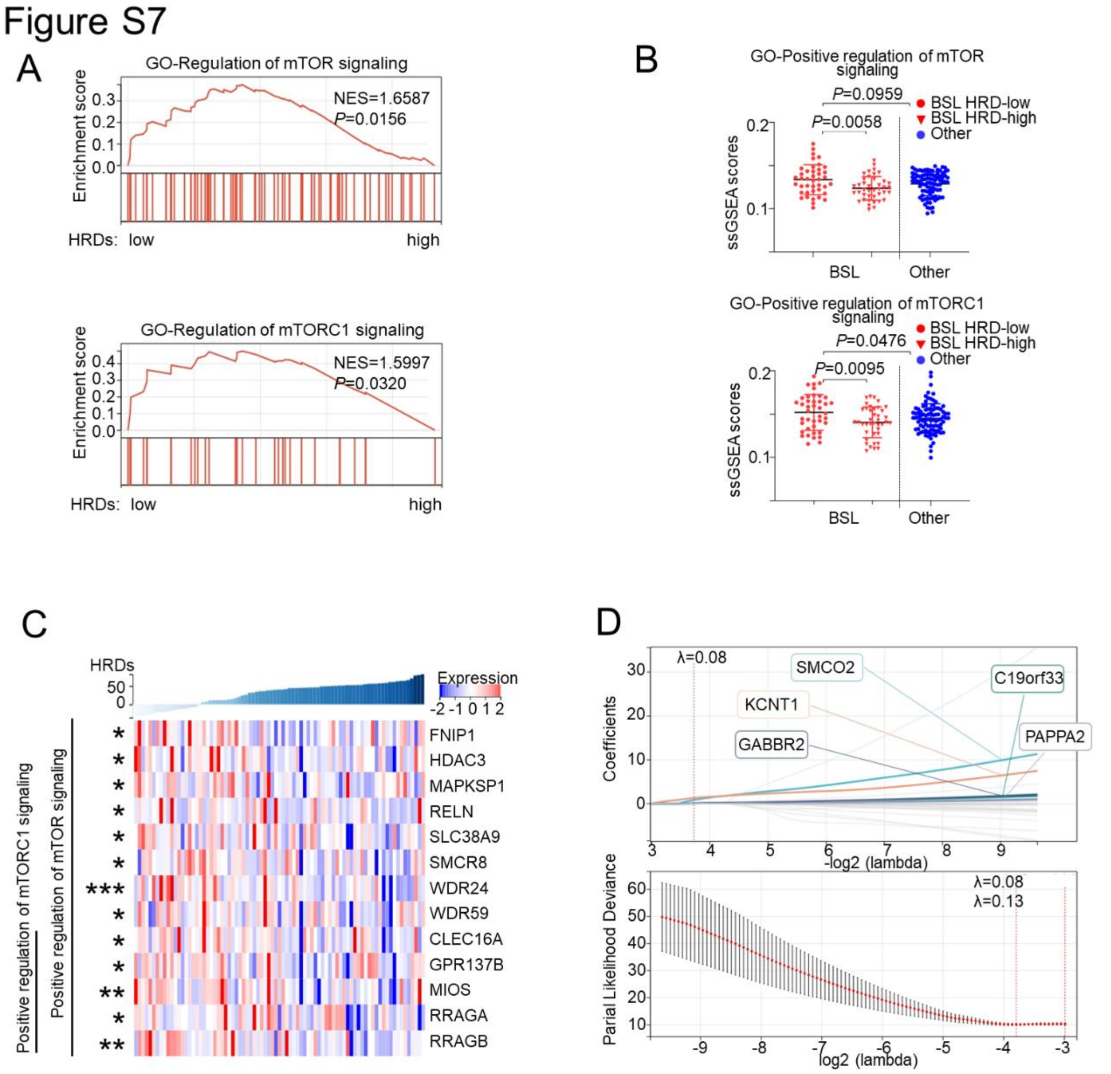

Supplementary Figure 7.

A, GSEA of regulation of mTOR signaling (top) and regulation of mTORC1 signaling (bottom) between BSL and other subgroups based on the GO dataset (permutation test). B, GO-Positive regulation of mTOR signaling (top) and mTORC1 signaling (bottom) GSEA scores among HRD-high, HRD-low BSL, and other subgroups (student's t test). C, Main genes of Positive regulation of mTOR signaling (total) and Positive regulation of mTORC1 signaling (bottom) in the BSL subgroup of HER2-low TNBC were shown in the heat-map. D, Lasso-Cox analysis on DEGs between bottom 25% and top 25% of HRDs in BSL. $P<0.05$ was considered as statistical significance. *, $P<0.05$. **, $P<0.01$.

***, P<0.001.

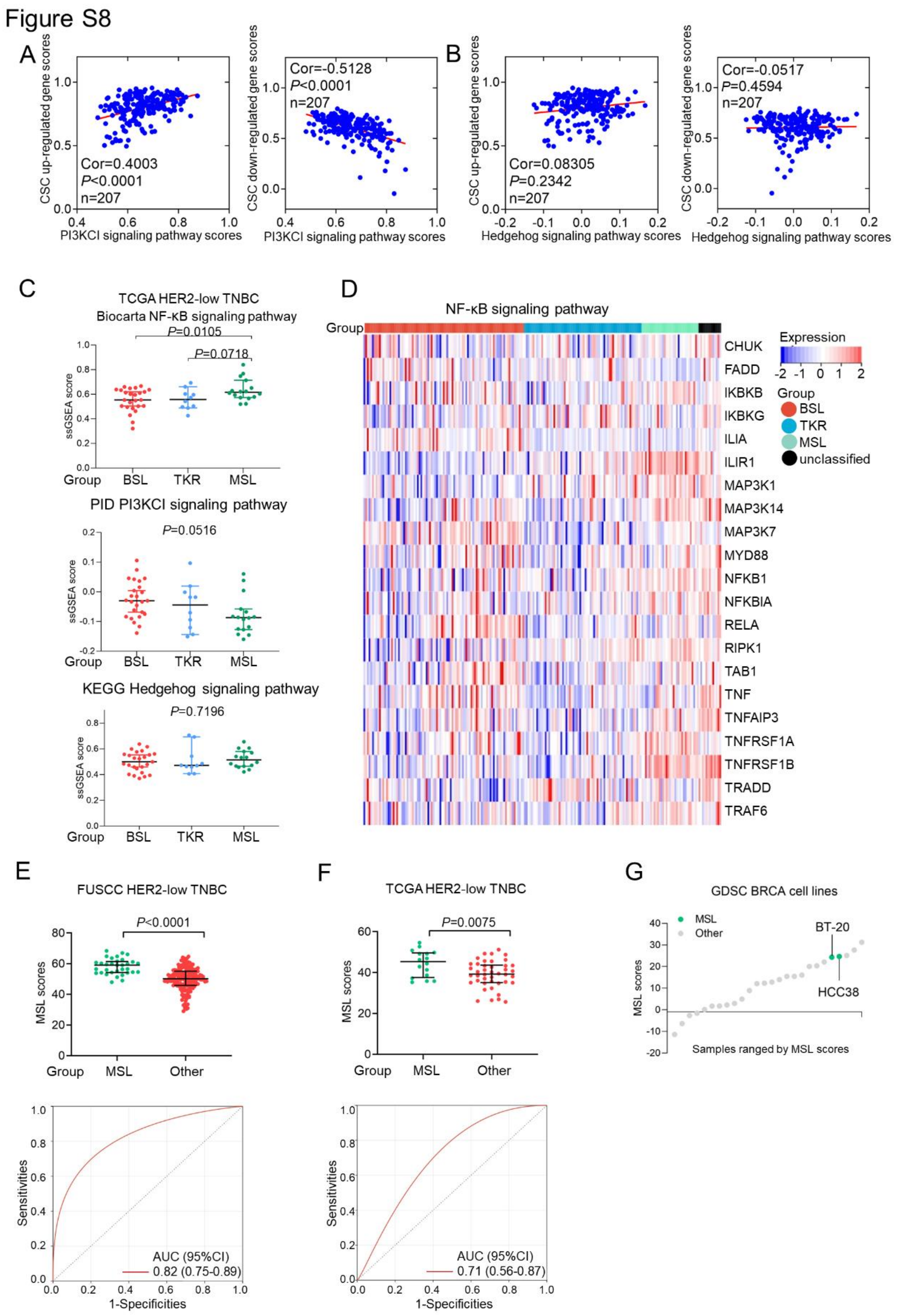

Supplementary Figure 8.

A, Spearman correlational analysis was performed between PI3KCI signaling pathway ssGSEA scores and CSC up-regulated gene scores (left), CSC down-regulated gene

scores (right) in the MSL subgroup. B, Spearman correlational analysis was performed between Hedgehog signaling pathway ssGSEA scores and CSC up-regulated gene scores (left), CSC down-regulated gene scores (right) in the MSL subgroup. C, ssGSEA scores of Biocarta-NF-κB signaling pathway (top), PID-PI3KCI signaling pathway (middle), and KEGG-Hedgehog signaling pathway (bottom) among BSL, TKR, and MSL subgroups from TCGA HER2-low TNBC (1-way ANOVA followed by Dunnett-t test). D, Expression of pivotal genes from NF-κB signaling pathway in HER2-low TNBC. E and F, MSL scores between MSL and other subgroups (top) and area AUC of ROC analysis of MSL scores to estimate the MSL subgroup (bottom) from HER2-low TNBC samples based on FUSCC (E) and TCGA (F) datasets, E (Mann-Whitney test), F (student's t test). G, MSL scores of BT-20, HCC-38, and other HER2-low breast cancer were illustrated based on the GDSC dataset. $P<0.05$ was considered as statistical significance.

Supplementary Table 1. Highlights of characteristics of HER2-low TNBC subgroups

Subgroup	BSL	TKR	MSL
Clinical	5-years RFS: 87.1%	5-years RFS: 89.7%;<br>high prevalence in Chinese	5-years RFS: 78.8%
Mutation	TP53 (62.5%);<br>low PI3KCA (5.4%);<br>low FOXA1 (0);<br>HRDs high	TP53 (48.7%);<br>high PI3KCA (43.6%);<br>high FOXA1 (10.3%)	TP53 (71.4%);<br>Middle PI3KCA (14.3%)<br>high FOXA1 (14.3%)
Activated pathway	HRD-low: mTOR and mTORC1 signaling	ERBB2 signaling;<br>receptor tyrosine kinase;<br>MAPK signaling	NF-κB signaling
Potential therapy	HRD-low: mTOR and mTORC1 signaling inhibitor	sequential Lapatinib or Tucatinib and DS-8201;<br>targeting MAPK signaling inhibitor	Bortezomib;<br>Targeting CSCs

BSL, basal like; TKR, receptor tyrosine kinase relevant; MSL, mesenchymal stem-like; RFS, relapse-free survival; HRD, homologous recombination deficiency.

DS-8201, antibody–drug conjugates (ADC).

Lapatinib and Tucatinib, the small-molecule tyrosine kinase inhibitors (TKIs).

Bortezomib, NF-κB pathway inhibitor.

Supplementary Materials and Methods

Dataset Collection and Data Processing

We constructed a Chinese triple-negative breast cancer (TNBC) dataset based on patients treated at Fudan University Shanghai Cancer Center (FUSCC) from January 2007 to December 2014. FUSCC dataset provided RNA-seq gene expression of 207 HER2-low TNBC samples (HER2 IHC 1+: n=140; IHC 2+, ISH-: n=67). Copy number alternations (CNVs), DNA-seq mutations, and clinical information of HER2-low breast cancer samples were also obtained. This study was approved by the Ethics Committee of FUSCC and each patient provided informed consent.

To validate our findings from FUSCC dataset, we used 58 HER2-low TNBC samples (IHC 1+: n=48; IHC 2+, ISH-: n=10) as well as 251 HR-positive and HER2-low cases (IHC 1+: 223; HER2 IHC 2+, ISH-: n=28) from the cancer genome atlas (TCGA, PanCancer Atlas, https://portal.gdc.cancer.gov/) dataset. We also gathered clinical information of breast cancer samples based on FUSCC and TCGA datasets. Phosphorylated protein expression of breast cancer samples was collected from the cancer proteome atlas (TCPA, https://www.tcpaportal.org/).

Genomics of Drug Sensitivity in Cancer (GDSC, https://www.cancerrxgene.org/) provides mRNA expressions of different kinds of breast cancer cells (1), while the MD Anderson Cell Lines Project (MCLP, https://www.tcpaportal.org/mclp) provide protein levels of breast cancer cells(2, 3).

Expression-based Unsupervised clustering

We utilized partitioning-around-medoid (PAM) clustering by ConsensusClusterPlus(4)

and consensus clustering to divide breast cancer samples from FUSCC into appropriate groups preliminarily. Two thousand most variable mRNAs (according to standard error, SD) were selected to construct breast cancer profiles, and the clustering robustness of PAM clustering (10 repetitions and 0.8 resamplings) was evaluated by consensus clustering. Consensus empirical cumulative distribution function (CDF) curves and consensus values were performed to determine optional groups. We repeated the above methods to regroup Cluster 4 (immune-related cluster). TCGA breast cancer data were grouped based on analogous methods.

Tumor-Infiltrating Lymphocytes (TILs) and Immune Cell Numbers Estimation

Both stromal TILs and intratumor TILs scores of breast cancer were obtained from FUSCC. CIBERSORT(5) (https://cibersort.stanford.edu/) created by Newman et al. was used to estimate the relative abundance of 22 immune cells (B cells memory, B cells naive, Macrophages M1, Macrophages M2, etc.) by deconvolution of the bulk RNA sequencing data in breast cancer from FUSCC and TCGA datasets.

Gene Set Enrichment Analysis (GSEA) and single sample GSEA (ssGSEA)

We performed GSEA utilizing GSEA software and the Molecular Signatures Database (http://www.gsea-msigdb.org/gsea/msigdb/). C2. Kyoto Encyclopedia of Genes and Genomes (KEGG) dataset, C5. GO dataset, Biocarta dataset, and PID dataset were selected to evaluate related pathways and molecular mechanisms [1000 repetitions, p-value < 0.05 or false discovery rate (FDR) < 0.25 were considered statistically significant]. The ssGSEA scores were calculated based on the R package "GSVA" and student's t test(6) was used to distinguish the difference between one group and the rest

(p-value < 0.05 was considered as statistical significance). Gene sets of relative pathways enrichment might suggest these pathways activation.

CNAs, Homologous Recombination Deficiency Score (HRDs), and DNA-seq mutation estimation

CNAs and DNA-seq mutation data were based on FUSCC and TCGA datasets. The three factors used to determine HRDs were genomic loss of heterozygosity (LOH), telomere allele imbalance (TAI), and large-scale state transition (LST)(7), and HRDs were also collected from FUSCC and TCGA dataset.

Constructing HRDR score system

For HRDR score, we firstly identified HRD-related (HRDR) DEGs by the Limma R package. The Glmnet R package was utilized to integrate RFS time, RFS status and HRD-related DEGs expression and Lasso-Cox method was utilized for regression analysis. HRDR score = 0.8418**SMCO2* expression + 0.0601**C19orf33* expression + 0.1103**GABBR2* expression +1.2934**KCNT1* expression + 0.0198**PAPPA2* expression.

Western Blotting

The specific primary antibodies were rabbit anti-HER2 antibody (1:1000, Cell Signaling Technology), rabbit anti-MAPK antibody (1:1000, Cell Signaling Technology), rabbit anti-p-MAPK (Thr202/Tyr204) antibody (1:1000, Cell Signaling Technology), rabbit anti-c-Myc antibody (1:1000, Cell Signaling Technology), mouse anti-Bcl-2 (1:1000, Cell Signaling Technology), rabbit anti-Bcl-xl antibody (1:1000, Cell Signaling Technology), mouse anti-Tubulin (1:1000, Cell Signaling Technology),

rabbit anti-GAPDH (1:1000, Cell Signaling Technology). HRP substrate (Millipore, USA) was used to detect HRP-conjugated secondary with an Image Quant LAS 4000 mini-imaging system (GE, Fairfield, USA).

Study approval

The use of clinical cancer samples was approved by the Ethics Committee of FUSCC (Protocol number: 050432-4-2108) and each patient signed informed consent. The animal model protocol was approved by the Institutional Animal Care and Use Committees (Number: FUSCC-IACUC-S2022-0245).

Reference:

1. Iorio F, et al. A Landscape of Pharmacogenomic Interactions in Cancer. *Cell.* 2016;166(3):740-54.
2. Chen MM, et al. Predicting Cancer Cell Line Dependencies From the Protein Expression Data of Reverse-Phase Protein Arrays. *JCO Clin Cancer Inform.* 2020;4:357-66.
3. Li J, et al. Characterization of Human Cancer Cell Lines by Reverse-phase Protein Arrays. *Cancer Cell.* 2017;31(2):225-39.
4. Wilkerson MD, and Hayes DN. ConsensusClusterPlus: a class discovery tool with confidence assessments and item tracking. *Bioinformatics.* 2010;26(12):1572-3.
5. Newman AM, et al. Robust enumeration of cell subsets from tissue expression profiles. *Nat Methods.* 2015;12(5):453-7.
6. Barbie DA, et al. Systematic RNA interference reveals that oncogenic KRAS-driven cancers require TBK1. *Nature.* 2009;462(7269):108-12.
7. Sztupinszki Z, et al. Migrating the SNP array-based homologous recombination deficiency measures to next generation sequencing data of breast cancer. *NPJ Breast Cancer.* 2018;4:16.
